# Can EQ-5D-3L utility values of low back pain patients be validly predicted by the Oswestry Disability Index for use in cost-effectiveness analyses?

**DOI:** 10.1007/s11136-022-03082-6

**Published:** 2022-01-17

**Authors:** Sylvia Pellekooren, Ângela J. Ben, Judith E. Bosmans, Raymond W. J. G. Ostelo, Maurits W. van Tulder, Esther T. Maas, Frank J. P. M. Huygen, Teddy Oosterhuis, Adri T. Apeldoorn, Miranda L. van Hooff, Johanna M. van Dongen

**Affiliations:** 1 Department of Health Sciences, Faculty of Science, Vrije Universiteit Amsterdam, Amsterdam Movement Sciences Research Institute, De Boelelaan 1105, 1081 HV Amsterdam, The Netherlands; 2 Department Human Movement Sciences, Faculty of Behavioral & Movement Sciences, Vrije Universiteit, Amsterdam, The Netherlands; 3 Department of Health Sciences, Faculty of Science, Vrije Universiteit Amsterdam, Amsterdam Public Health Research Institute, Amsterdam, The Netherlands; 4 Department of Epidemiology and Data Science, Amsterdam UMC, Location VUmc, Amsterdam Movement Sciences Research Institute, Amsterdam, The Netherlands; 5 Center of Pain Medicine Erasmusmc, Rotterdam, The Netherlands; 6 Center of Pain Medicine UMCU, Utrecht, The Netherlands; 7 Netherlands Society of Occupational Medicine, Centre of Excellence, Utrecht, the Netherlands; 8 Coronel Institute of Occupational Health, Amsterdam UMC, University of Amsterdam, Amsterdam Public Health Research Institute, Amsterdam, The Netherlands; 9 Rehabilitation Departement, Noordwest Ziekenhuisgroep, Alkmaar, Netherlands, Breederode Hogeschool, Rotterdam, Netherlands; 10 Departement Research, Sint Maartenskliniek, Nijmegen, The Netherlands; 11 Department of Orthopedic Surgery, Radboud University Medical Center, Nijmegen, The Netherlands

**Keywords:** Low back pain, Utility scores, Oswestry Disability Index, Ordinary least squares, Tobit, EQ-5D

## Abstract

**Purpose:**

To assess whether regression modeling can be used to predict EQ-5D-3L utility values from the Oswestry Disability Index (ODI) in low back pain (LBP) patients for use in cost-effectiveness analysis.

**Methods:**

EQ-5D-3L utility values of LBP patients were estimated using their ODI scores as independent variables using regression analyses, while adjusting for case-mix variables. Six different models were estimated: (1) Ordinary Least Squares (OLS) regression, with total ODI score, (2) OLS, with ODI item scores as continuous variables, (3) OLS, with ODI item scores as ordinal variables, (4) Tobit model, with total ODI score, (5) Tobit model, with ODI item scores as continuous variables, and (6) Tobit model, with ODI item scores as ordinal variables. The models’ performance was assessed using explained variance (*R*^2^) and root mean squared error (RMSE). The potential impact of using predicted instead of observed EQ-5D-3L utility values on cost-effectiveness outcomes was evaluated in two empirical cost-effectiveness analysis.

**Results:**

Complete individual patient data of 18,692 low back pain patients were analyzed. All models had a more or less similar *R*^2^ (range 45–52%) and RMSE (range 0.21–0.22). The two best performing models produced similar probabilities of cost-effectiveness for a range of willingness-to-pay (WTP) values compared to those based on the observed EQ-5D-3L values. For example, the difference in probabilities ranged from 2 to 5% at a WTP of 50,000 €/QALY gained.

**Conclusion:**

Results suggest that the ODI can be validly used to predict low back pain patients’ EQ-5D-3L utility values and QALYs for use in cost-effectiveness analyses.

**Supplementary Information:**

The online version contains supplementary material available at 10.1007/s11136-022-03082-6.

## Plain English summary

Quality-Adjusted Life Years (QALY) are an essential outcome in economic evaluations that assess whether a new intervention is cost-effective (i.e., provides good value for money) compared to an alternative intervention. However, not all economic evaluations among low back pain patients measure quality of life using a preference-based measure which is necessary to calculate QALY. If a preference-based quality-of-life measure is missing, utility values may be predicted using other measurement instruments, such as a condition-specific questionnaire on low back pain complaints, such as the frequently used Oswestry Disability Index. However, it is unclear whether this results in valid estimates of the utility values. Therefore, we developed six different models that predicted utility values based on the Oswestry Disability Index and assessed their predictive ability. Additionally, we assessed the extent to which cost-effectiveness outcomes differed between the predicted and actual utility values for the EQ-5D-3L. Results suggest that the ODI can be validly used to predict low back pain patients’ QALYs in an economic evaluation when preference-based quality-of-life data are missing.

## Introduction

Low back pain (LBP) has an estimated incidence of 250 million people worldwide and is characterized by a high burden of disease [1]. Patients with LBP typically experience difficulties in different aspects of health-related quality of life, such as their daily functioning, social participation [2, 3], and working ability [4, 5]. These difficulties may affect patients’ health-related quality of life considerably [3, 6] and have significant impact on healthcare and societal costs [7, 8]. As limited (healthcare) resources are available, decision-makers are not only interested in the effectiveness of LBP treatments recommended in international guidelines, but also in their cost-effectiveness compared to alternative treatments.

Cost-effectiveness analysis provide insight into relative cost-effectiveness of treatments by comparing their incremental costs to their incremental effects [9]. These effects are often expressed in Quality-Adjusted Life Years (QALYs), which combine both the quality and quantity of life into a single outcome [10]. For estimating QALYs, health-related quality of life is typically measured using preference-based quality-of-life measures. Health states obtained from these measures can be converted into utility values, which represent the preferences of the general population of a country for given health states [11]. In many countries, it is recommended to estimate utility values using the EuroQol five-dimension questionnaire (EQ-5D) and national tariffs to account for the fact that health state preferences differ across countries [12–14]. Unfortunately, EQ-5D data are not always available in clinical trials [15], as higher priority is sometimes given to condition-specific measures that assess more clinically relevant outcomes [16].

When utility values are missing, QALYs cannot be calculated. However, information about incremental cost per QALY gained is typically required by healthcare decision-makers, particularly at the national level [12, 13]. In the absence of the EQ-5D or another generic preference-based quality-of-life measure, a condition-specific measure might be used to predict utility values [17]. In LBP, one of the most frequently used condition-specific measures is the Oswestry Disability Index (ODI) [18]. The ODI measures limitations of a patient’s performance [19] and is recommended in the core outcome set for clinical trials in nonspecific LBP [20] and management of LBP [21].

A previous study assessed the predictive ability of the ODI in estimating utility values from the EQ-5D-3L by using data from 14,544 patients with lumbar degenerative pathology treated in a tertiary spine center [22]. Linear regression analysis was performed to predict the patients’ EQ-5D utility values based on their ODI total or individual item scores and patients reported severity of back and leg pain. Based on a root mean square error (RMSE) of 0.14, authors concluded that it is not possible to estimate EQ-5D-3L utility values based on the ODI. However, given the bounded nature of EQ-5D data as well as the possible existence of other contextual factors that influence health-related quality of life in LBP, it is likely that the models’ performance might be improved by using a Tobit model to account for possible ceiling effects. The model’s performance might also be improved by including a wider variety of LBP patients treated in various settings, while adjusting for more case-mix variables. Moreover, the authors only based their conclusions on the models’ RMSE without assessing the impact of using predicted utility scores in cost-effectiveness. Therefore, this study aimed to assess the feasibility of using different regression models to predict EQ-5D-3L utility values in LBP patients based on the ODI in cost-effectiveness analyses while adjusting for a broad range of case-mix characteristics.

## Method

### Source of data

Individual patient data included in this study originated from four previously conducted prospective studies; i.e., the minimal interventional treatments (MINT) study, the rehabilitation after lumbar disk surgery (REALISE) study, the Nijmegen Decision Tool study, and a study evaluating a treatment-based classification system [23–32]. These studies were conducted among sub-acute and chronic LBP patients treated in primary care, secondary care, and/or tertiary care. For all patients, various sociodemographic variables were assessed at baseline, and both the ODI and EQ-5D-3L utility values were assessed at baseline and at one or more follow-up moments. In total, 21,500 patients were included in these studies. For developing the models, only baseline data were used in the present study, because the proportion of participants with missing data was low at baseline (i.e., < 5%), thereby preventing the need for imputation of missing values. To assess the final models’ performance in a trial-based cost-effectiveness analysis setting, baseline as well as follow-up data were used of the MINT study [23–25], and the treatment-based classification system study [29, 30].

The MINT study [23–25], the REALISE study [31, 32], and the treatment-based classification system study [29, 30] obtained ethical approval from the Medical Ethics Committee of the Erasmus Medical Centre Rotterdam or Medical Ethics Committee of the VU University Medical Centre in Amsterdam. For the Nijmegen Decision Tool study [26–28], ethical approval was not required, because the “*Dutch Act on Medical Research involving Human Subjects*” does not apply to screening questionnaires that are part of routine practice. More detailed information on the design and study population of the different studies is provided in Supplementary Appendix A.

### Utility values

Utility values were based on the EQ-5D-3L, which is a generic preference-based measure that asks participants to describe their health state on five health dimensions (i.e., mobility, self-care, usual activities, pain/ discomfort, and anxiety/depression) using three severity levels (i.e., no problems, moderate problems, and severe problems)[33]. The participants’ EQ-5D-3L health states were converted into utility values using the Dutch tariff[34]. Utility values are presented on a continuous scale that is anchored at 1 (indicating full health) to 0 (indicating a state as bad as being dead). Negative values may also occur, which represent health states that are regarded as worse than a state that is as bad as being dead [10]. Dutch EQ-5D-3L utility values can range between − 0.33 and 1.

### Oswestry Disability Index

The ODI measures the limitations of a patient’s performance compared with that of a fit person, and consists of ten items assessing various aspects of daily living (e.g., lifting, walking, and traveling). Each item is scored on a six-point scale, ranging from 0 to 5. The overall ODI score was estimated by summing the values of all individual items, subsequently dividing this score by the total possible score, and multiplying this score by 100. The total score ranges from 0 to 100%, with higher scores indicate higher level of disability [19, 35]. For this study, the “sex life” (item 8) was not included, as this item is frequently omitted in applied studies as well [36–38]. Including this item would have hampered the generalization of the results to a large number of LBP studies. The cross cultural adapted Dutch language version of the ODI version 2.1a was used in all studies included [39].

### Predictors

The following case-mix variables were included; age (years), gender (male/female), education level (low/moderate/high), living together with a partner (yes/no), type of LBP (sub-acute/chronic), setting (primary care/secondary care/ tertiary care), and back pain (Numeric Rating Scale (NRS: 0–10) Pain score: low 0–3, moderate 4–6, and severe 7–10) [40]. Given error proneness of overly detailed models and benefits of ease of use, NRS scores were categorized using cut-off points from an earlier conducted study, which categorized NRS pain scores based on pain-related interference with functioning in patients with chronic musculoskeletal pain [41]. These variables were included, because they were expected to increase the predictive value of the models [42–47] and to be measured in most applied studies, thereby increasing applicability of the models.

### Statistical analysis

Baseline characteristics were described using frequencies and percentages for categorical variables and means and standard deviations for continuous variables. Prior to the development of the models, linearity and additivity assumptions (i.e., normally distributed residuals, homoscedasticity, influential cases and outliers) were assessed using diagnostic plots (i.e., scatterplot, density plot, and boxplots), and diagnostic tests (e.g., Grubbs test). Pearson’s correlation coefficient was used to assess the strength of the linear relationship between the patients’ EQ-5D-3L based utility values and ODI total scores. To assess the agreement between the EQ-5D-3L and the ODI the Intra Class Correlation (ICC)was calculated using a two-way random effects model.

### Model development and variable selection

Models were developed using two regression techniques; i.e., Ordinary Least Squares (OLS) regression and Tobit regression (i.e., censored or truncated regression). OLS regression was included, because it is still one of the most frequently used linear modeling techniques. OLS regression is used to estimate the strength of the association between a continuous outcome variable and one or more independent variables [48]. OLS, however, does not take into account the bounded nature of utility values which can be accounted for in a Tobit regression [49]. This model can estimate linear relationships between variables, where the range of the dependent variable is constrained. This is done using a so-called latent variable that accounts for the fact that the true independent variable is—in our case—bounded at 1. Hereby, biased and inconsistent estimates, that may occur when using OLS regression, may be prevented [50].

For both the OLS and Tobit model, three different regression models were developed: (1) including the overall ODI score as independent variable, (2) using all nine ODI items scores as independent variables and assuming them to be continuous, and (3) using all nine ODI items scores as independent variables and assuming them to be ordered. This resulted in six different models: (1) OLS, with the total ODI score, (2) OLS, with the ODI item scores as continuous variables, (3) OLS, with the ODI item scores as ordinal variables, (4) Tobit model, with the total ODI score, (5) Tobit model, with the ODI item scores as continuous variables, (6) Tobit model, with the ODI item scores as ordinal variables. To assess which variables increased the predictive value of the models, a bi-directional stepwise selection procedure [51], using Akaike Information Criterion (i.e., the trade-off between the goodness of fit of the model and the simplicity of the model) [52], with a 5% significance level was used. Stepwise selection combines the elements of forward and backward selection by sequentially adding variables, based on the most contributing predictors, and omitting variables that no longer provide an improvement in the model fit after adding a new variable to the model. Final models only included case-mix variables that increased the predictive value.

### Model performance and internal validation

The original dataset was split into a training sample (70%), and a validation sample (30%) using the ‘create Data Partition’ function in R. This function creates a balanced split of the data by performing a stratified random split of the data based on the mean of the dependent variable, which leads to a comparable mean EQ-5D-3L utility value in both the training and validation dataset. After developing the models in the training sample, their performance was assessed in the validation sample using the RMSE (i.e., the absolute fit of the model) and the adjusted *R*^2^ (i.e., the relative fit of the model). The minimal important difference (MID) of the EQ-5D-3L was used to determine an acceptable RSME, which was set at a cut of point of 0.03 [53]. A correlation of 0.5 or higher (i.e., a relatively moderate correlation as the R squared indicates that about half of the variance of the utility values is explained by the ODI) was considered sufficient for performing regression analysis. Recommended models were selected based on parsimony, which is the trade-off between simplicity of the model (i.e., low AIC) and explanatory predictive power (i.e., high *R*^2^). To assess agreement between the actual and estimated EQ-5D-3L based utility values a Bland Altman analysis was performed for all models.

### Sensitivity analyses

In addition to the main analysis, three sensitivity analyses (SA) were performed. In the first sensitivity analysis (SA1) the variable mental health status was added to the case-mix variables (SA1). SA1 was only performed on a sub-set of the data, as only one of the four datasets (i.e., the MINT study [23–25]) assessed mental health using the Four Dimensional Symptom Questionnaire (4DSQ) [53], and only part of the sample (*n* = 4123) completed this questionnaire. The 4DSQ assesses four different aspects of mental health (i.e., distress, depression, anxiety, and somatisation), all of which were included in the models as a separate variable. In SA2, the variable living with a partner was omitted. In SA3 the patients’ EQ-5D-3L utility values were converted to EQ-5D-5L utility values using the reverse crosswalk (SA3) [55]. Reversed cross walk values make it possible to link EQ-5D-3L responses to EQ-5D-5L value sets, and can be used when 5L values are wanted, but only 3L data are available [55, 56]. The 5-level EQ-5D version is an adapted version of the EQ-5D-3L, which is known to be more sensitive and has less ceiling effects, including through changing the number of levels of perceived problems per dimension from 3 to 5[57].

### Cost-effectiveness analysis

To assess the models’ impact on cost-effectiveness outcomes, complete cases from two randomized controlled trials were used, i.e., empirical dataset 1 (*n* = 68; Apeldoorn et al. [29, 30]) and empirical dataset 2 (*n* = 424; Maas et al. [23–25]). In both studies, QALYs were estimated based on both the actual EQ-5D-3L scores (i.e., actual QALY values) and based on the patients’ ODI scores (i.e., predicted QALY values). Agreement between the actual and estimated EQ-5D-3L based utility values was assessed by performing a Bland Altman analysis for each of the empirical datasets.

Then, full trial-based cost-effectiveness analysis were conducted for each of the six models as well as the patients’ actual QALY values (i.e., QALYs based on the measured EQ-5D-3L scores). For each trial-based cost-effectiveness analysis, mean differences in costs and QALYs between treatment groups were estimated using seemingly unrelated regression analyses. Incremental Cost-Effectiveness Ratios (ICERs) were calculated by dividing the difference in costs by the difference in effects. Uncertainty around cost and QALY differences was estimated using bootstrapping. The percentage of bootstrapped cost-effect pairs was reported per quadrant of the Cost-Effectiveness Plane (i.e., north east, south east, north west, and south west). Subsequently, Cost-Acceptability Curves (CEACs) were plotted. CEACs indicate an intervention’s probability of cost-effectiveness compared to control for a range of willingness-to-pay (WTP) values (i.e., thresholds of 0, 30,000 euro and 50,000). These probabilities were assessed on their decision sensitivity (i.e., how sensitive is the conclusion of a cost-effectiveness analysis is to using a particular statistical method) [58] . Analyses were performed in R software, version 3.4.0.

## Results

### Participants

Out of the individual patient data that included 21,500 patients, 18,692 complete cases were included for analysis. These patients had sub-acute (*n* = 3248) or chronic LBP (*n* = 15,444). The mean age of the patients was 53.9 years (SD = 14.7, range 18.1–91.9) and 61% of the sample was female. The patients’ mean ODI score at baseline was 41.23 (SD = 15.4, range 0–100) and their mean baseline EQ-5D-3L based utility value was 0.46 (SD = 0.29, range -0.3290–1.00). More details on the patients’ characteristics are shown in Table 1.

**Table 1:** Baseline characteristics of patients included

Characteristic	*n* = 18,692
Age (mean (SD), range)	53.9 (14.7), 18.1–91.9
Gender; female (*n*, %)	11,345 (60.7)
Education (*n*, %)	
Low (no education, primary level education, lower vocational and lower secondary education)	5,398 (28.9)
Moderate (higher secondary education or undergraduate)	9,078 (48.6)
High (tertiary, university level, postgraduate)	4,216 (22.6)
Living with a partner (*n*, %)	14,085 (75.4)
Type of LBP (*n*, %)	
Sub-acute (< 3 months)	3,248 (17.4)
Chronic (> 3 months)	15,444 (82.6)
Post-surgery (*n*, %)	1,587 (8.5)
Setting (*n*, %)	
Primary care (i.e., physiotherapy clinics)	150 (0.8)
Secondary care (i.e., pain clinics)	4,123 (22.1)
Tertiary care (i.e., hospital)	14,419 (77.1)
NRS pain (mean (SD))	6.99 (1.9)
Utility score (mean (SD), range)	0.467 (0.299), − 0.3290–1.00
ODI score^a^ (mean (SD), range)	41.23 (15.4), 0–100
ODI 1 mean (SD)/median (IQR)	2.66 (0.93)/3 (2–4)
ODI 2 mean (SD)/median (IQR)	1.11 (1.04)/1 (0–2)
ODI 3 mean (SD)/median (IQR)	2.78 (1.32)/3 (2–4)
ODI 4 mean (SD)/median (IQR)	1.44 (1.22)/1 (0–2)
ODI 5 mean (SD)/median (IQR)	2.11 (1.09)/2 (1–3)
ODI 6 mean (SD)/median (IQR)	2.85 (1.29)/3 (2–4)
ODI 7 mean (SD)/median (IQR)	1.49 (1.09)/1 (0–2)
ODI 9 mean (SD)/median (IQR)	2.14 (1.20)/2 (1–3)
ODI 10 mean (SD)/median (IQR)	1.98 (1.32)/2 (1–3)

^a^Excluding item 8 sex life

*LBP* low back pain, *NRS* numeric rating scale (range 0–10), utility (range − 0.33 to 1), *ODI* oswestry disability scale (range 0–100), ODI individual item (range 0–5), *SD* standard deviation, *IQR* inter quartile range

### Variables included and model performance

The diagnostic plots showed a linear relationship between EQ-5D-3L based utility values and the ODI, and homogeneity of variance of the residuals. Even though the patients’ baseline EQ-5D-3L based utility values followed a bimodal distribution, the corresponding residuals were normally distributed. Hence, the normality of residuals assumption of linear regression was met. No outliers or influential cases were identified. Pearson’s correlation coefficient between the patients’ baseline EQ-5D-3L utility values and ODI total score was 0.63. The ICC showed an agreement of 0.23 between individual ODI items and EQ-5D-3L items.

An overview of the independent variables that were included in the final models, as well as their respective regression coefficients, can be found in Supplementary Appendix B. The case-mix variables age, gender, education, partner, and NRS were included in all models, whereas type of LBP was not included in any of the models. The variable setting was included in all models except for model 1 (i.e., OLS with ODI total scores). In the models using Tobit regression, 74 of the 13,087 observations in the training set were right censored.

The performance of the different models was more or less the same, with explained variances ranging from 45 to 51% and RMSEs ranging from 0.21 to 0.22. Based on parsimony of the models, model 2 and 5 seem most appropriate to use. More details on the performance of the different models are shown in Table 2.

**Table 2:** Performance measures in the training set

	Performance in the training set (*n* = 13,087)		AIC	Performance in validation set (*n* = 5605)		AIC
	R^2^	RMSE		R^2^	RMSE	
Model 1: OLS with ODI total scores	0.45	0.22	− 2326.48	0.46	0.22	− 1083.26
Model 2: OLS with ODI individual item total scores continuous	0.50	0.21	− 3423.24	0.50	0.21	− 1513.73
Model 3: OLS with ODI individual item total scores ordered	0.51	0.21	− 3769.51	0.52	0.21	− 1638.09
Model 4: Tobit with ODI total scores	0.45	0.22	− 2061.91	0.46	0.22	− 951.61
Model 5: Tobit with ODI individual item total scores continuous	0.50	0.21	− 3164.37	0.50	0.21	− 1385.32
Model 6 Tobit with individual item total scores ordered	0.51	0.21	− 3474.88	0.52	0.21	− 1494.06

*OLS* ordinary least squares regression, *ODI* oswestery disability index, *R*^2^ proportion of variance for the dependent variable, *RMSE* root mean squared error, *AIC* akaike information criteria

**Fig. 1:**
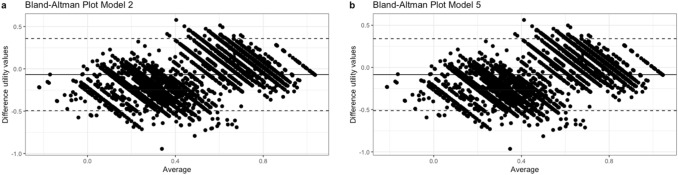
Bland Altman plot model 2 and 5. *X*-axis: average measurement of the estimated and actual utility values, *Y*-axis: difference in measurements between the two instruments. Solid line: Average difference in measurements between the estimated and actual utility values, Dashed lines: 95% confidence interval limits for the average difference

The mean difference between estimated and actual utility values for model 2 was -0.068 (95%CI -0.495, 0.359), and for model 5 -0.086 (95%CI -0.512, 0.341). Bland Altman plots of models 2 and 5 are shown in Fig. 1. The plots for other all models are presented in Supplementary Appendix C.

### Sensitivity analysis

Adding mental health variable(s) to the models resulted in an increase of the explained variance of 2–4%, whereas the RMSE remained similar. Omission of the variable ‘living with a partner’ (SA2) did not change the models’ performance. Using the patients’ reversed cross-walked EQ-5D-5L utility values (SA3) improved the models’ explained variance by 3–4%, and the RMSE reduced with 0.06–0.07. More details on the results of the sensitivity analyses are provided in Supplementary Appendix D.

### Results cost-effectiveness analysis

**Fig. 2:**
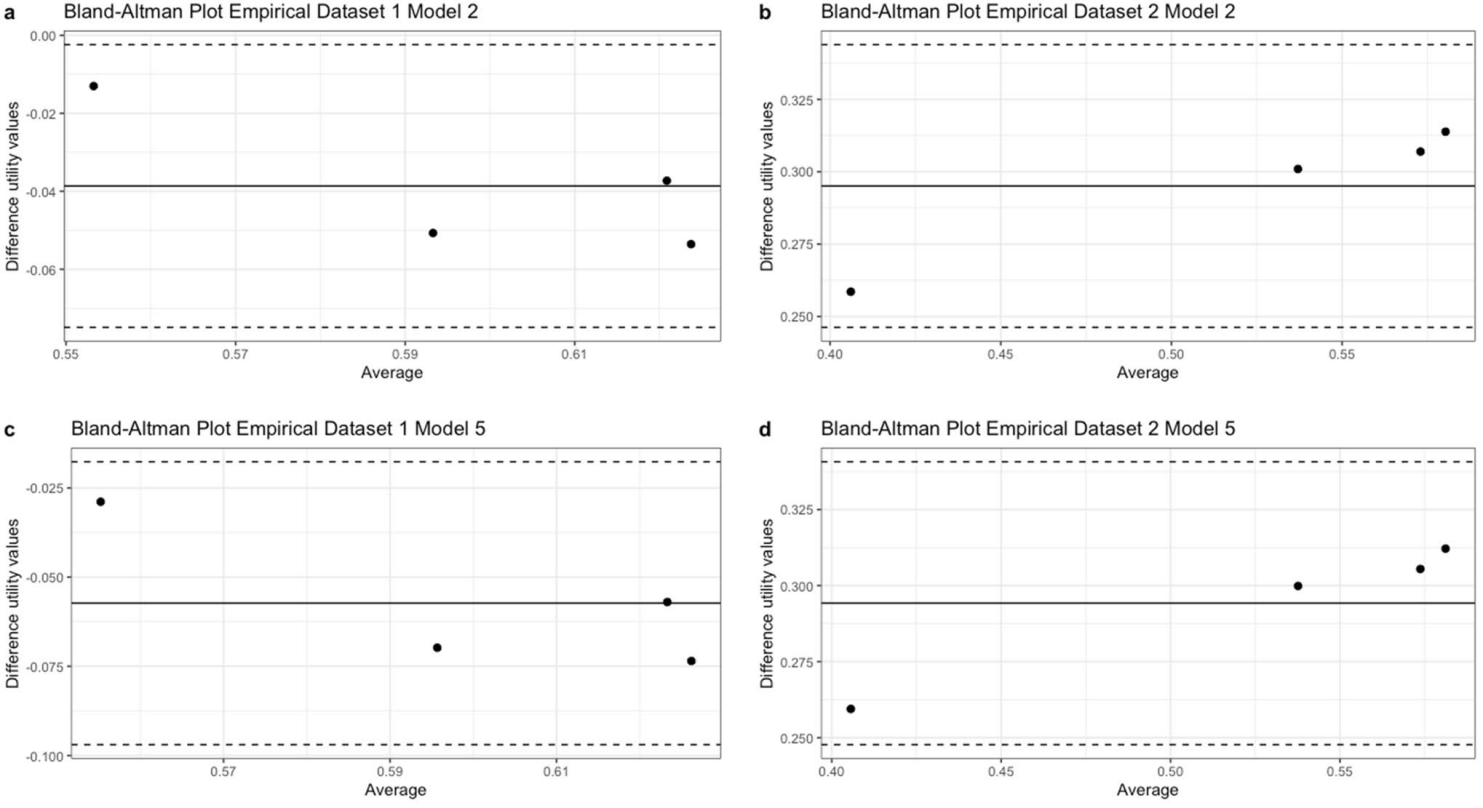
Bland Altman plot model 2 and 5 empirical datasets. *X*-axis: average measurement of the estimated and actual utility values, *Y*-axis: difference in measurements between the two instruments. Solid line: average difference in measurements between the estimated and actual utility values, Dashed lines: 95% confidence interval limits for the average difference

The mean difference between estimated and actual utility values for empirical dataset 1 model 2 was -0.039 (95%CI -0.075, -0.002), and for model 5 -0.057 (95%CI -0.097, -0.018). The mean difference between estimated and actual utility values for empirical dataset 2 model 2 was 0.295 (95%CI 0.246, 0.344), and for model 5 the mean difference was 0.294 (95%CI 0.248, 0.341). Bland Altman plots of models 2 and 5 for both empirical datasets are shown in Fig. 2. The plots for other all models are presented in Supplementary Appendix E.

**Fig. 3:**
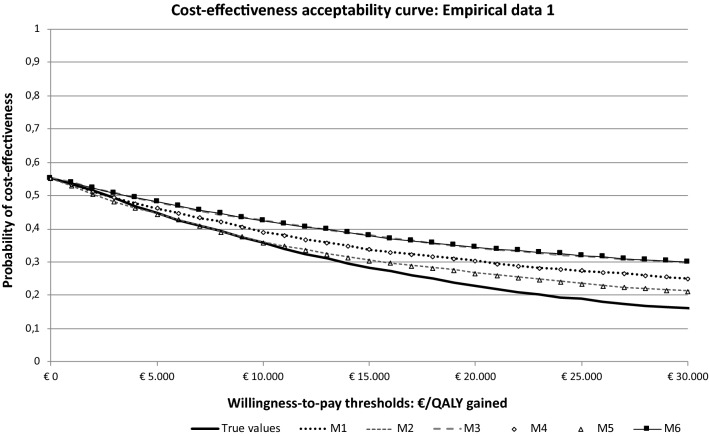
Cost-effectiveness acceptability curves empirical dataset 1. *M1* model 1, *M2* model 2, *M3* model 3, *M4* model 4, *M5* model 5, *and M6* model 6

In both empirical datasets, the difference between the predicted and actual differences in QALYs was small for the two most parsimonious models (i.e., models 2 and 5:∆ ≤ 0.004) and the distributions of cost-effect pairs across the four quadrants of the cost-effectiveness plane were comparable. The cost-effectiveness acceptability curves based on both predicted and actual QALY values were also similar. The predicted probability of an intervention being cost effective at a willingness to pay of 50,000 was slightly higher in both models than the actual probabilities (i.e., 2–5% in model 2, and 3–5% in model 5). More details on the cost-effectiveness outcomes for all models in both empirical studies are shown in Table 3 and Fig. 3.

**Table 3:** Cost-effectiveness outcomes for an intervention in comparison with usual care by predictive models

Predictive models	Δ*E* (95% CI)	Δ*C* (95% CI)	ICER	Cost-effectiveness plane				Cost-effectiveness acceptability curve			
				NE (%)	SE (%)	SW (%)	NW (%)	*P*_CE_ (0)	*P*_CE_ (10,000)	*P*_CE_ (30,000)	*P*_CE_ (50,000)
Empirical dataset 1 [28, 29]* N* = 86											
Actual values	− 0.041 (− 0.091; 0.009)	− 110 (− 1761; 1283)	2697	2	4	51	42	0.55	0.36	0.16	0.11
Model 1	− 0.035 (− 0.094; 0.021)	− 110 (− 1761; 1283)	3091	1	10	45	44	0.55	0.39	0.25	0.20
**Model 2**	− **0.043 (**− **0.106; 0.015)**	− **110 (**− **1761; 1283)**	**2559**	**1**	**7**	**48**	**44**	**0.55**	**0.36**	**0.21**	**0.16**
Model 3	− 0.027 (− 0.081; 0.018)	− 110 (− 1761; 1283)	4068	1	13	43	43	0.55	0.42	0.30	0.24
Model 4	− 0.036 (− 0.095; 0.021)	− 110 (− 1761; 1283)	3058	1	10	45	44	0.55	0.39	0.25	0.20
**Model 5**	− **0.044 (**− **0.107; 0.015)**	− **110 (**− **1761; 1283)**	**2514**	**1**	**7**	**48**	**44**	**0.55**	**0.36**	**0.21**	**0.16**
Model 6	− 0.027 (− 0.080; 0.021)	− 110 (− 1761; 1283)	4084	2	13	42	43	0.55	0.42	0.30	0.25
Empirical dataset 2 [22–24] * N* = 424											
Actual values	− 0.004 (− 0.034; 0.027)	1576 (596; 2575)	− 371,566	38	0	0	62	0.001	0.002	0.017	0.048
Model 1	− 0.007 (− 0.037; 0.023)	1576 (596; 2575)	− 226,441	32	0	0	68	0.001	0.002	0.014	0.037
**Model 2**	**0.0002 (**− **0.030; 0.029)**	**1576 (596; 2575)**	**6,670,132**	**51**	**0**	**0**	**49**	**0.001**	**0.003**	**0.025**	**0.070**
Model 3	− 0.001 (− 0.026; 0.024)	1576 (596; 2575)	− 2,099,247	48	0	0	52	0.001	0.002	0.015	0.028
Model 4	− 0.007 (− 0.037; 0.024)	1576 (596; 2575)	− 224,080	32	0	0	67	0.001	0.002	0.014	0.038
**Model 5**	**0.0003 (**− **0.030; 0.030)**	**1576 (596; 2575)**	**5,105,447**	**51**	**0**	**0**	**49**	**0.001**	**0.003**	**0.025**	**0.073**
Model 6	− 0.001 (− 0.027; 0.026)	1576 (596; 2575)	− 2,417,793	48	0	0	51	0.001	0.002	0.018	0.053

Recommended models are presented as bold text

*N* number of observations in the analysis, *ΔC* difference in costs, *95% CI* 95% confidence interval. *ΔE* difference in effects, *ICER* incremental cost-effectiveness ratio, *NE* north east, *SE* south east, *SW* south west, *NW* north west, *P*_*CE*_* (0)* probability that the intervention is cost-effective as compared to usual care with a threshold of 0, *P*_*CE*_* ( )* probability that the intervention is cost-effective as compared to usual care with willingness-to-pay thresholds of 0, 10,000, 30,000, and 50,000 Euros

## Discussion

### Main findings

There were no large differences in the models’ performance between OLS and Tobit regression, nor between using the patients’ total ODI scores and ODI individual item scores. The explained variance of the developed models ranged from 45 to 51%, and the RMSE ranged from 0.21 to 0.22. Models 2 and 5 are recommended based on the best fit and parsimony. The models’ relatively low absolute fit (RMSE) indicates that they are not suitable for estimating utility values for individual patients. Nonetheless, they can be used to predict differences in LBP patients’ EQ-5D-3L utility values and QALY’s, as the systematic bias in mean scores does not affect the differences between the groups. Cost-effectiveness outcomes of models 2 and 5 based on predicted and actual values were similar. These findings enable researchers to perform a cost-effectiveness analysis with QALYs as the outcome measure, even if EQ-5D-3L data are missing.

### Comparison with literature

Our findings regarding the performance measures are more or less in line with the previous study by Carreon et al. [20], who aimed to predict individual LBP patients’ EQ-5D-3L utility values based on their ODI scores. Their model performed slightly better in terms of its explained variance (i.e., R^2^ was 61%) and its absolute fit (i.e., RMSE is 0.149), which is probably the result of a more homogenous study population, and therefore may indicate an overfitting of their model. Based on the RMSE, Carreon et al. [20] concluded that individual patients’ EQ-5D-3L utility values could not validly be predicted from their ODI scores. Although we agree with this conclusion, we would like to stress that a low RMSE does not necessarily mean that the models cannot be used in the context of an cost-effectiveness analysis. This is true, when the bias surrounding the predicted utility values does not translate into relevant differences in incremental QALYs and the probability of the intervention being cost-effective compared to the control group (i.e., decision-based validity) [58]. This may be explained by the fact that the bias is likely to be similar in the intervention and control groups, thereby not affecting incremental QALYs and CEACs [59].

### Strengths and limitations

To develop the models, a large sample of LBP patients from various settings (i.e., primary, secondary, and tertiary care) and with various complaint durations (i.e., subacute and chronic LBP) was used, which increases both the reliability and generalisability of the models. Moreover, next to OLS models, Tobit models were used to account for the constrained range of utility values [49, 50]. Although the added value of the Tobit model in this LBP population turned out to be rather limited, this might be different for LBP populations with milder symptoms, in which a larger share of patients is expected to report full health (i.e., a utility value of 1).

Our study also had some limitations. First, part of the sample was derived from two RCTs. Although RCT data may have limited generalisability, we chose to add these RCTs to our sample to create a more diverse sample and provide a better representation of the LPB population. Second, during the analysis, balanced data splitting was used to create the training and validation set. Although this balanced split provides better distribution of data then a random split, it might have been more appropriate to use K-fold cross validation[60]. Unfortunately, running the Tobit model using *k*-fold cross validation was not feasible as the R package for the Tobit model was not compatible with the *K*-fold package. In a post hoc analysis we developed and validated the OLS models with *k*-fold cross validation and this produced similar results as our main analysis (data not shown). We also expect this to be the case for the Tobit models. Third, EQ-5D-3L utilities were used instead of EQ-5D-5L utilities. This is a limitation because EQ-5D-5L is known to be more sensitive and therefore recommended in pharmacoeconomic guidelines. Nonetheless, some countries still use the EQ-5D-3L. Therefore, we preferred to use the current relatively large dataset with EQ-5D-3L utility values of nearly 20,000 patients for developing and validating the models, instead of using a relatively small dataset with EQ-5D-5L. As the performance measures in the sensitivity analysis using the EQ-5D-5L reversed cross walk were comparable with those of the EQ-5D-3L version, we expect that EQ-5D-5L values can also be validly estimated using ODI scores. Fourth, the models were based on Dutch utility values. Previous research has shown that there are differences in utilities, QALYs, ICERs, and CEACs between countries due to the use of different value sets per country [14]. Therefore, we added the regression coefficients of models 2 and 5 for different countries in Supplementary Appendix F. These regression coefficients are based on the available value sets (tariffs) for different countries, and can be used to calculate utility values and QALYs. Fifth, some data that were used were to assess the performance of the developed models in a trial-based cost-effectiveness analysis setting were also part of the training set. However, as this was only a small percentage of the total training set (3.1%), we do not expect it to have influenced the validity of our finding that the difference between the estimated and true QALYs is small. Last, for assessing the performance of the developed models in a trial-based cost-effectiveness analysis setting, we only used data of two clinical trials, both of which found the intervention far from being cost-effective. That is, the probability of the interventions being cost-effective was low regardless of the willingness-to-pay threshold. In datasets where the interventions’ cost-effectiveness is less conclusive, even small differences in the probability of an intervention being cost-effective might impact the overall conclusion of a study. Further research in the form of a simulation study, using simulated data to examine the generalisability beyond the datasets, is needed to assess the performance of the developed models in a wide range of trial-based cost-effectiveness analysis settings.

### Implications for research and practice

Our findings suggest that predictive modeling can be used to estimate utility values from disease-specific measures, such as the ODI among LBP patients, when assessing incremental costs per QALY gained (as part of a cost-effectiveness analysis) or differences in utilities between groups. This is helpful for assessing cost-effectiveness in trials that did not directly measure utilities. Given the relatively large RMSE (i.e., low absolute fit of the models) and the relatively low r-square value (i.e., low relative fit) it is strongly discouraged to use the developed models to estimate the utility values of individual patients. Further research is needed to validate the models in order to (1) assess whether these models yield comparable results in other empirical datasets on LBP interventions, especially in analysis on interventions that are expected not to be conclusive in their cost-effectiveness, and (2) to improve their generalisability among different LBP patients by external validation in another sample. This study focused on assessing the validity of predictive regression modeling in estimating EQ-5D-3L utility values from the ODI and the impact of these estimated utility values on cost-effectiveness analysis. Results show that this is feasible for estimating QALYs and ICERs, but not for estimating individual utility scores. Further research is needed to explore whether other mapping methods, such as response mapping approaches like non-parametric and multinomial logistic regression [16, 54, 55], result in better predictive accuracy in estimating individual utility values of preference-based measures, such as the EQ-5D. This is important because studies suggest these mapping methods might be better at preventing regression to the mean [61]. Additional research might not only result in more accurate estimated utility values, but would also provide insight into the relative performance of different methods to estimate these values.

In the meantime, researchers can use the developed models in their cost-effectiveness analysis when utility values are lacking. Of them, the OLS model (i.e., model 2) is recommended in samples in which only a small number of patients has a utility value of 1 at baseline or follow-up measurement, whereas the Tobit model (i.e., model 5) is recommended in samples in which a substantial part of the sample has a utility score at baseline or at follow-up measurement. Although it seems possible to estimate utility values from disease-specific measures it is important to stress that it is still preferred to use preference-based quality-of-life measurements when setting up new studies.

## Conclusion

Results of this study suggest that the ODI can be used to predict LBP patients’ EQ-5D-3L utility values when the aim is to perform an cost-effectiveness analysis for QALYs, if utility values are missing, meaning in order to compare difference between groups of patients. The models are not suitable for estimating utility values for individual patients. Further research is needed to validate the models in order to assess whether these models yield comparable results in other empirical datasets on LBP interventions, to improve generalisability of the estimated models, and to compare the performance of predictive modeling compared to a mapping approach for estimating utility values. In the meantime, researchers can use the developed models in their cost-effectiveness analysis when utility values are lacking.

## Supplementary Information

Below is the link to the electronic supplementary material.

Electronic supplementary material 1 (DOCX 12568 kb)

## Data Availability

Data are available through the corresponding author upon reasonable request.

## Code availability

Codes are available through the corresponding author upon reasonable request.
