# Supplementary material for: Can EQ-5D-3L utility values of low back pain patients be validly predicted by the Oswestry Disability Index for use in cost-effectiveness analyses?
Source: Qual Life Res. 2022 Jan 17;31(7):2153–65. doi: 10.1007/s11136-022-03082-6 (PMC9188530; doi:10.1007/s11136-022-03082-6)
Supplement: Supplementary file 1 — Electronic supplementary material 1 (DOCX 12568 kb) [file 11136_2022_3082_MOESM1_ESM.docx]

**APPENDIX A. DESCRIPTION STUDIES INCLUDED**

**MINT Study**

The MINT study [23-25] assessed the effectiveness of radiofrequency denervation added to a standardized exercise program for patients with chronic low back pain. This study included patients with chronic LBP, receiving conservative treatment in a multidisciplinary pain clinic. This study was conducted at 16 multidisciplinary pain clinics in the Netherlands, and had both a randomized and observational track. The randomized track consisted of three sub trails, namely the facet joint trial, the sacroiliac joint trial, and the combination trial (facet joint, sacroiliac joint, or the intervertebral disk). Patients were consecutively screened, and were eligible when meeting the following criteria; a. pain considered to be related to the facet joint, sacroiliac joint, or a combination of the facet joint, sacroiliac joint, or intervertebral disk, aged 18 to 70 years, and no improvement in symptoms after conservative treatment. A total of 681 patients were included in the three randomized trails. Patients who were not willing to participate, or did not meet the inclusion criteria, were approached for the observational track of this study. In total 5168 patients were included in the observational track. Exclusion criteria for all trials were pregnancy, severe psychological problems, involvement in work-related conflicts or claims; body mass index higher than 35; or anticoagulant drug therapy or coagulopathy. Data was collection through surveys. For more details we refer to the original publications.

**Nijmegen Decision Tool Study**

In the Nijmegen Decision Tool Study (NDT study) [26-27] 47 indicators for a successful treatment outcome were assessed among chronic low back pain patients (CLBP), in order to compile a decision-support screening tool (NDT-CLBP) [28]. Patients were recruited at a Dutch orthopaedic hospital specialized in spine care, prior to their first consultation at the orthopaedic outpatient department. All consecutive low back pain patients were asked to complete the web-based questionnaire, which is part of routine practice. In total 14,859 patients with chronic LBP were included in this dataset. Patients were eligible when meeting the following criteria; experienced low back pain complaints for more than three months (i.e., CLBP) due to degenerative lumbar spine disorders (excluding trauma and tumor), had access to the internet, and were able to read and write Dutch. For more details we refer to the original publications.

**Study of Apeldoorn et al.**

The study of Apeldoorn et al. [29, 30] assessed the cost-effectiveness of a modified version of Delitto’s classification-based treatment approach compared with usual physical therapy care in patients with sub-acute and chronic LBP. This study included 156 patients with subacute and chronic LBP treated in a primary care setting. Patients were recruited by during their first contact with a physical therapist working in the region of Amsterdam. Patients were eligible when meeting the following criteria; LBP as the primary complaint (with or without associated leg pain), age between 18 and 65 years, current episode longer than 6 weeks, and able to read and write Dutch. Exclusion criteria were known- or suspected-specific LBP, severe radiculopathy, serious co-morbidity and psychopathology. Data was collection through surveys. For more details we refer to the original publications.

**REALISE Study**

The REALISE study [31-32] concerned the assessment of effectiveness, and cost effectiveness of referral for early rehabilitation after lumbar disc surgery. This multicentre, randomised, controlled trial included 169 LPB patients with a herniated lumbar disc postoperatively treated in a primary care facility. Patients were referred to the research team by neurosurgeons, and checked on eligibility by research nurses. Patients were eligible when meeting the following criteria; a herniated lumbar disc confirmed by magnetic resonance imaging (MRI) and signs of nerve root compression corresponding to the level of disc herniation, aged between 18 and 70 years, and were able to fill out questionnaires in Dutch themselves. Exclusion criteria were cauda equina syndrome, neurogenic claudication, co-morbidities of the lumbar spine, spinal surgery in the prior 12 months, contraindications to exercise therapy, pregnancy, or previous lumbar disc surgery at the same level and on the same side. Data was collection through surveys. For more details we refer to the original publications.

**TABLE A1. BASELINE CHARACETRISTICS INCLUDED STUDIES (COMPLETE CASES EQ-5D and ODI)**

|  | **Apeldoorn Study [29, 30]**  **n=156** | **MINT Study [23-25]**  **n=6,316** | **Nijmegen study [26-28]**  **n=14,859** | **REALISE Study [31-32]**  **n=169** |
| --- | --- | --- | --- | --- |
| Age (years), mean (SD) | 42.5 (11.2) | 56.2 (13.5) | 53.5 (15.1) | 47.3 (11.8) |
| Sex, n (%) |  |  |  |  |
| Female | 89 (57.1) | 3,576 (67.1) | 8,695 (58.5) | 98 (58.0) |
| Male | 67 (42.9) | 1,757 (32.9) | 6,164 (41.5) | 71 (42.0) |
| Education level, n (%) |  |  |  |  |
| Low (no education, primary level education, lower vocational and lower secondary education) | 27 (17.3) | 1,892 (29.9) | 3,922 (26.4) | 37 (21.9) |
| Moderate (higher secondary education or undergraduate) | 61 (39.1) | 2,406 (38.1) | 6,967 (47.8) | 97 (57.4) |
| High (tertiary, university level, postgraduate) | 68 (43.6) | 823 (13.03) | 3,403 (22.9) | 35 (20.7) |
| Living together with a partner, n (%) |  |  |  |  |
| Yes | 119 (76.3) | 4,663 (73.8) | 11,118 (74.8) | 125 (74.0) |
| No | 37 (23.7) | 1,593 (25.2) | 3,741 (25.2) | 44 (26.0) |
| Type of low back pain, n (%) |  |  |  |  |
| Subacute (< 3 months) | 32 (20.5) | 3,601 (57.0) | 423 (2.8) | 0 |
| Chronic (> 3 months) | 124 (79.5) | 1,682 (26.6) | 14,436 (97.2) | 169 (100.0) |
| Post-surgery, n (%) |  |  |  |  |
| Yes | 0 | 0 | 0 | 169 (100.0) |
| No | 156 (100.0) | 6,316 (100.0) | 14,859 (100.0) | 0 |
| Setting, n (%) |  |  |  |  |
| Primary care (i.e., physiotherapy clinics) | 156 (100.0) | 0 | 0 | 169 (100.0) |
| Secondary care (i.e., pain clinics) | 0 | 6,316 (100.0) | 0 | 0 |
| Tertiary care (i.e., hospital) | 0 | 0 | 14,859 (100.0) |  |
| NRS Pain, mean (SD) | 6.1 (1.8) | 7.3 (1.6) | 6.9 (2.0) | 6.3 (2.6) |
| Utility, mean (SD) | 0.7 (0.2) | 0.5 (0.3) | 0.5 (0.3) | 0.4 (0.3) |
| ODI, mean (SD) | 20.6 (13.0) | 39.6 (14.6) | 42.0 (15.4) | 31.1 (14.3) |
| SD = Standard Error, NRS = Numeric Rating Scale range 0-10, Utility range: -0.33 – 1, ODI: Oswestery Disability Index range: 0-100 | | | | |

**APPENDIX B. REGRESSION COEFFICIENTS MODEL 1-6**

| **Model 1. Ordinary Least Squares Regression with ODI total scores**  Utility = 0.833 - 0.011*ODI total score + 0.002*age + 0.012*female + 0.015 *education middle + 0.021 *education high - 0.014 *no partner + 0.015* NRS moderate - 0.115 *NRS severe | | | |
| --- | --- | --- | --- |
| Regression Coefficient (SE) | | 95% CI | |
|  |  | 2.5 % | 97.5 % |
| Intercept | 0.833 (0.016) | 0.807 | 0.857 |
| ODI total score | -0.011 (0.000) | -0.011 | -0.010 |
| Age | 0.002 (0.000) | 0.001 | 0.002 |
| Gender; female | 0.012 (0.004) | 0.003 | 0.019 |
| Education; middle | 0.015 (0.004) | 0.006 | 0.024 |
| Education; high | 0.021 (0.006) | 0.010 | 0.032 |
| Partner; no partner | -0.014 (0.005) | -0.023 | -0.005 |
| NRS; moderate | 0.015 (0.008) | -0.001 | 0.031 |
| NRS; severe | -0.115 (0.009) | -0.131 | -0.098 |

| **Model 2. Ordinary Least Squares Regression with ODI individual items scores (continuous)**  Utility = 0.936 - 0.095*ODI1 - 0.044*ODI2 - 0.005*ODI3 - 0.019*ODI4 - 0.004*ODI5 - 0.008*ODI6 - 0.014*ODI7 - 0.033*ODI9 - 0.019*ODI10 + 0.002*age + 0.008*female + 0.019*education middle + 0.026*education high - 0.014*no partner - 0.066* secondary care - 0.051*tertiary care + 0.034*NRS moderate - 0.062*NRS severe | | | |
| --- | --- | --- | --- |
|  | | 95% CI | |
|  | Regression Coefficient (SE) | 2.5 % | 97.5 % |
| Intercept | 0.936 (0.024) | 0.889 | 0.984 |
| ODI1 | -0.095 (0.003) | -0.099 | -0.089 |
| ODI2 | -0.044 (0.002) | -0.049 | -0.039 |
| ODI3 | -0.005 (0.002) | -0.009 | -0.002 |
| ODI4 | -0.019 (0.002) | -0.023 | -0.015 |
| ODI5 | -0.004 (0.002) | -0.008 | -0.000 |
| ODI6 | -0.008 (0.002) | -0.012 | -0.005 |
| ODI7 | -0.014 (0.002) | -0.018 | -0.010 |
| ODI9 | -0.033 (0.002) | -0.039 | -0.029 |
| ODI10 | -0.019 (0.002) | -0.023 | -0.015 |
| Age | 0.002 (0.000) | 0.001 | 0.002 |
| Gender; female | 0.008 (0.004) | 0.000 | 0.015 |
| Education; middle | 0.019 (0.005) | 0.009 | 0.027 |
| Education; high | 0.026 (0.005) | 0.016 | 0.037 |
| Partner; no partner | -0.014 (0.004) | -0.023 | -0.006 |
| Setting; secondary care | -0.066 (0.022) | -0.108 | -0.024 |
| Setting; tertiary care | -0.051 (0.021) | -0.093 | -0.009 |
| NRS; moderate | 0.034 (0.008) | 0.019 | 0.049 |
| NRS; severe | -0.062 (0.008) | -0.079 | -0.045 |

| **Model 3. Ordinary Least Squares Regression with ODI individual items scores (ordered)**  Utility = 0.794 + 0.020*ODI1;1 - 0.004*ODI1;2 -0.138*ODI1;3 - 0.246*ODI1;4 - 0.247*ODI1;5 - 0.053 *ODI2;1 + 0.006*ODI2;2 - 0.106*ODI2;3 - 0.190*ODI2;4 - 0.146*ODI2;5 + 0.001*ODI3;1 - 0.001*ODI3;2 - 0.006*ODI3;3 - 0.012*ODI3;4 - 0.039*ODI3;5 - 0.017*ODI4;1 - 0.033*ODI4;2 -0.048*ODI4;3 - 0.069*ODI4;4 - 0.131*ODI4;5 + 0.004*ODI5;1 + 0.006*ODI5;2 - 0.009*ODI5;3 - 0.016*ODI5;4 - 0.026*ODI5;5 - 0.001*ODI6;1 - 0.005*ODI6;2 - 0.011*ODI6;3 - 0.024*ODI6;4 - 0.043*ODI6;5 - 0.003*ODI7;1 - 0.023*ODI7;2 - 0.036 *ODI7;3 - 0.049*ODI7;4 - 0.051*ODI7;5 - 0.024*ODI9;1 - 0.034*ODI9;2 - 0.093*ODI9;3 - 0.154*ODI9;4 - 0.153*ODI9;5 - 0.020*ODI10;1 - 0.042*ODI10;2 - 0.060*ODI10;3 - 0.079*ODI10;4 - 0.066*ODI10;5 + 0.002*age + 0.007*female + 0.017*education middle + 0.026*education high - 0.013*no partner - 0.088*secondary care - 0.078*tertiary care + 0.002*NRS moderate - 0.080*NRS severe | | | |
| --- | --- | --- | --- |
|  | | 95% CI | |
|  | Regression Coefficient (SE) | 2.5 % | 97.5 % |
| Intercept | 0.794 (0.028) | 0.738 | 0.849 |
| ODI1;1 | 0.020 (0.018) | -0.015 | 0.055 |
| ODI1;2 | -0.004 (0.017) | -0.037 | 0.029 |
| ODI1;3 | -0.138 (0.017) | -0.172 | -0.104 |
| ODI1;4 | -0.246 (0.018) | -0.281 | -0.211 |
| ODI1;5 | -0.247 (0.022) | -0.291 | -0.204 |
| ODI2;1 | -0.053 (0.005) | -0.063 | -0.043 |
| ODI2;2 | 0.006 (0.006) | -0.109 | -0.087 |
| ODI2;3 | -0.106 (0.008) | -0.122 | -0.089 |
| ODI2;4 | -0.190 (0.015) | -0.221 | -0.160 |
| ODI2;5 | -0.146 (0.040) | -0.224 | -0.070 |
| ODI3;1 | 0.001 (0.010) | -0.019 | 0.020 |
| ODI3;2 | -0.001 (0.010) | -0.021 | 0.020 |
| ODI3;3 | -0.006 (0.010) | -0.025 | 0.013 |
| ODI3;4 | -0.012 (0.010) | -0.032 | 0.008 |
| ODI3;5 | -0.039 (0.013) | -0.064 | -0.013 |
| ODI4;1 | -0.017 (0.005) | -0.026 | -0.007 |
| ODI4;2 | -0.033 (0.006) | -0.045 | -0.021 |
| ODI4;3 | -0.048 (0.007) | -0.062 | -0.033 |
| ODI4;4 | -0.070 (0.010) | -0.088 | -0.050 |
| ODI4;5 | -0.131 (0.028) | -0.186 | -0.077 |
| ODI5;1 | 0.004 (0.008) | -0.012 | 0.020 |
| ODI5;2 | 0.006 (0.008) | -0.009 | 0.021 |
| ODI5;3 | -0.009 (0.009) | -0.025 | 0.008 |
| ODI5;4 | -0.016 (0.010) | -0.036 | 0.005 |
| ODI5;5 | -0.026 (0.018) | -0.061 | 0.008 |
| ODI6;1 | -0.001 (0.011) | -0.022 | 0.021 |
| ODI6;2 | -0.005(0.011) | -0.027 | 0.016 |
| ODI6;3 | -0.011(0.011) | -0.032 | 0.011 |
| ODI6;4 | -0.024 (0.011) | -0.045 | -0.003 |
| ODI6;5 | -0.043(0.013) | -0.070 | -0.017 |
| ODI7;1 | -0.003(0.006) | -0.015 | 0.009 |
| ODI7;2 | -0.023(0.007) | -0.036 | -0.010 |
| ODI7;3 | -0.036(0.008) | -0.108 | -0.078 |
| ODI7;4 | -0.049(0.013) | -0.074 | -0.025 |
| ODI7;5 | -0.051(0.016) | -0.082 | -0.020 |
| ODI9;1 | -0.024(0.007) | -0.039 | -0.009 |
| ODI9;2 | -0.034(0.007) | -0.048 | -0.019 |
| ODI9;3 | -0.093(0.008) | -0.108 | -0.078 |
| ODI9;4 | -0.154(0.011) | -0.175 | -0.132 |
| ODI9;5 | -0.153(0.011) | -0.187 | -0.119 |
| ODI10;1 | -0.020(0.008) | -0.037 | -0.004 |
| ODI10;2 | -0.042(0.009) | -0.060 | -0.024 |
| ODI10;3 | -0.060(0.010) | -0.079 | -0.040 |
| ODI10;4 | -0.079 (0.011) | -0.101 | -0.057 |
| ODI10;5 | -0.066(0.012) | -0.090 | -0.041 |
| Age | 0.002(0.000) | 0.001 | 0.002 |
| Gender; female | 0.007(0.004) | -0.001 | 0.014 |
| Education; middle | 0.017(0.004) | 0.008 | 0.026 |
| Education; high | 0.026(0.005) | 0.015 | 0.036 |
| Partner; No partner | -0.013(0.004) | -0.021 | -0.004 |
| Setting; secondary care | -0.088 (0.022) | -0.131 | -0.046 |
| Setting; tertiary care | -0.078 (0.022) | -0.120 | -0.035 |
| NRS; moderate | 0.002 (0.008) | -0.014 | 0.018 |
| NRS; severe | -0.080 (0.009) | -0.096 | -0.063 |

| **Model 4. Tobit with ODI total scores**  Utility = 0.897 - 0.011*ODI total score + 0.002*age + 0.011*female + 0.015*education middle + 0.021*education high - 0.014*no partner - 0.058*secondary care - 0.058*tertiary care + 0.010*NRS moderate - 0.119*NRS severe | | | |
| --- | --- | --- | --- |
|  | | 95% CI | |
|  | Regression Coefficient (SE) | 2.5 % | 97.5 % |
| Intercept | 0.897 (0.025) | 0.848 | 0.947 |
| ODI total score | -0.011 (0.000) | -0.011 | -0.011 |
| Age | 0.002 (0.000) | 0.002 | 0.002 |
| Gender; female | 0.011 (0.004) | 0.004 | 0.020 |
| Education; middle | 0.015 (0.004) | 0.006 | 0.024 |
| Education; high | 0.021 (0.006) | 0.010 | 0.032 |
| Partner; no partner | -0.014 (0.005) | -0.023 | -0.005 |
| Setting; secondary care | -0.058 (0.023) | -0.104 | -0.013 |
| Setting; tertiary care | -0.058 (0.023) | -0.103 | -0.013 |
| NRS; moderate | 0.010 (0.008) | -0.006 | 0.026 |
| NRS; severe | -0.119 (0.009) | -0.136 | -0.102 |

| **Model 5. Tobit with ODI individual items scores (continuous)**  Utility = 0.961 - 0.096*ODI1 - 0.044*ODI2 - 0.005*ODI3 - 0.019*ODI4 - 0.005*ODI5 - 0.009*ODI6 - 0.014*ODI7 - 0.033*ODI9 - 0.019*ODI10 + 0.002*age + 0.008*female + 0.018*education middle + 0.026* education high - 0.014*no partner - 0.079*secondary care - 0.064* tertiary care + 0.029*NRS moderate - 0.066*NRS severe | | | |
| --- | --- | --- | --- |
|  | | 95% CI | |
|  | Regression Coefficient (SE) | 2.5 % | 97.5 % |
| Intercept | 0.961 (0.025) | 0.913 | 1.009 |
| ODI1 | -0.096 (0.003) | -0.101 | -0.091 |
| ODI2 | -0.044 (0.002) | -0.048 | -0.040 |
| ODI3 | -0.005 (0.002) | -0.009 | -0.002 |
| ODI4 | -0.019 (0.002) | -0.023 | -0.015 |
| ODI5 | -0.005 (0.002) | -0.008 | -0.000 |
| ODI6 | -0.009 (0.002) | -0.012 | -0.005 |
| ODI7 | -0.014 (0.002) | -0.018 | -0.010 |
| ODI9 | -0.033 (0.002) | -0.037 | -0.029 |
| ODI10 | -0.019 (0.002) | -0.023 | -0.015 |
| Age | 0.002 (0.000) | 0.001 | 0.002 |
| Gender; female | 0.008 (0.004) | 0.000 | 0.016 |
| Education; middle | 0.018 (0.005) | 0.009 | 0.027 |
| Education; high | 0.026 (0.005) | 0.016 | 0.037 |
| Partner; no partner | -0.014 (0.004) | -0.023 | -0.006 |
| Setting; secondary care | -0.079 (0.022) | -0.123 | -0.036 |
| Setting; tertiary care | -0.064 (0.022) | -0.107 | -0.021 |
| NRS; moderate | 0.029 (0.008) | 0.013 | 0.045 |
| NRS; severe | -0.066 (0.009) | -0.083 | -0.049 |

| **Model 6. Tobit with ODI individual items scores (ordered)**  Utility = 0.831 - 0.006*ODI1;1 - 0.019*ODI1;2 - 0.153*ODI1;3 - 0.261*ODI1;4 - 0.262*ODI1;5 - 0.053*ODI2;1 - 0.098*ODI2;2 - 0.105*ODI2;3 - 0.190*ODI2;4 - 0.146*ODI2;5 - 0.002*ODI3;1 - 0.030*ODI3;2 - 0.009*ODI3;3 - 0.014*ODI3;4 - 0.041*ODI3;5 - 0.017*ODI4;1 - 0.033*ODI4;2 - 0.048*ODI4;3 - 0.069*ODI4;4 - 0.132*ODI4;5 - 0.004*ODI5;1 - 0.006*ODI5;2 - 0.009*ODI5;3 - 0.016*ODI5;4 -0.027*ODI5;5 - 0.002*ODI6;1 - 0.007*ODI6;2 - 0.013*ODI6;3 - 0.029*ODI6;4 - 0.045*ODI6;5- 0.003*ODI7;1 - 0.023*ODI7;2 - 0.036*ODI7;3 - 0.050*ODI7;4 - 0.051*ODI7;5 - 0.026*ODI9;1 - 0.036*ODI9;2 - 0.095*ODI9;3 - 0.155*ODI9;4 - 0.155*ODI9;5 -0.022*ODI10;1 - 0.043*ODi10;2 - 0.061*ODI10;3 - 0.080*ODI10;4 - 0.067*ODI10;5 + 0.0015134*age + 0.017 *education middle + 0.026* education high - 0.013*no partner - 0.099* secondary care - 0.089*tertiary care + 0.000 *NRS moderate - 0.081*NRS severe | | | |
| --- | --- | --- | --- |
|  | | 95% CI | |
|  | Regression Coefficient (SE) | 2.5 % | 97.5 % |
| Intercept | 0.831 (0.029) | 0.774 | 0.888 |
| ODI1;1 | 0.006 (0.018) | -0.029 | 0.042 |
| ODI1;2 | -0.019 (0.017) | -0.054 | 0.015 |
| ODI1;3 | -0.153 (0.018) | -0.188 | -0.120 |
| ODI1;4 | -0.261 (0.018) | -0.297 | -0.226 |
| ODI1;5 | -0.262 (0.023) | -0.306 | -0.218 |
| ODI2;1 | -0.053 (0.005) | -0.063 | -0.043 |
| ODI2;2 | -0.098 (0.006) | -0.109 | -0.087 |
| ODI2;3 | -0.105 (0.008) | -0.122 | -0.089 |
| ODI2;4 | -0.190 (0.016) | -0.220 | -0.159 |
| ODI2;5 | - 0.146 (-0.040) | -0.224 | -0.069 |
| ODI3;1 | -0.002 (0.010) | -0.021 | 0.018 |
| ODI3;2 | -0.030 (0.010) | -0.024 | 0.018 |
| ODI3;3 | -0.009 (0.010) | -0.028 | 0.011 |
| ODI3;4 | -0.014 (0.010) | -0.034 | 0.006 |
| ODI3;5 | -0.041 (0.013) | -0.066 | -0.016 |
| ODI4;1 | -0.017 (0.005) | -0.027 | -0.007 |
| ODI4;2 | -0.033 (0.006) | -0.045 | -0.021 |
| ODI4;3 | -0.048 (0.007) | -0.063 | -0.037 |
| ODI4;4 | -0.069 (0.010) | -0.088 | -0.051 |
| ODI4;5 | -0.132 (0.028) | -0.187 | -0.078 |
| ODI5;1 | -0.004 (0.008) | -0.012 | 0.020 |
| ODI5;2 | -0.006 (0.008) | -0.010 | 0.021 |
| ODI5;3 | -0.009 (0.009) | -0.026 | 0.008 |
| ODI5;4 | -0.016 (0.011) | -0.037 | 0.004 |
| ODI5;5 | -0.027 (0.018) | -0.061 | 0.008 |
| ODI6;1 | -0.002 (0.011) | -0.023 | 0.020 |
| ODI6;2 | -0.007 (0.011) | -0.029 | 0.015 |
| ODI6;3 | -0.013 (0.011) | -0.034 | 0.090 |
| ODI6;4 | -0.029 (0.011) | -0.047 | -0.004 |
| ODI6;5 | -0.045 (0.014) | -0.071 | -0.019 |
| ODI7;1 | -0.003 (0.006) | -0.015 | 0.008 |
| ODI7;2 | -0.023 (0.007) | -0.036 | -0.010 |
| ODI7;3 | -0.036 (-0.008) | -0.051 | -0.021 |
| ODI7;4 | -0.050 (0.013) | -0.074 | -0.025 |
| ODI7;5 | -0.051 (0.016) | -0.083 | -0.020 |
| ODI9;1 | -0.026 (0.008) | -0.041 | -0.011 |
| ODI9;2 | -0.036 (0.008) | -0.050 | -0.021 |
| ODI9;3 | -0.095 (0.008) | -0.110 | -0.080 |
| ODI9;4 | -0.155 (0.011) | -0.177 | -0.133 |
| ODI9;5 | -0.155 (0.017) | -0.189 | -0.121 |
| ODI10;1 | -0.022 (0.009) | -0.038 | -0.005 |
| ODI10;2 | -0.043 (0.009) | -0.062 | -0.025 |
| ODI10;3 | -0.061 (0.010) | -0.081 | -0.041 |
| ODI10;4 | -0.080 (-0.011) | -0.103 | -0.058 |
| ODI10;5 | -0.067 (0.012) | -0.091 | -0.042 |
| Age | 0.002 (0.000) | 0.001 | 0.002 |
| Gender; female | -0.007 (0.004) | -0.001 | 0.015 |
| Education; middle | 0.017 (0.004) | 0.008 | 0.025 |
| Education; high | 0.026 (0.005) | 0.015 | 0.036 |
| Partner; no partner | -0.013 (0.004) | -0.021 | -0.004 |
| Setting; secondary care | -0.099 (0.022) | -0.143 | -0.056 |
| Setting; tertiary care | -0.089 (0.022) | -0.132 | -0.046 |
| NRS; moderate | 0.000 (0.008) | -0.016 | 0.016 |
| NRS; severe | -0.081 (0.009) | -0.098 | -0.064 |

**APPENDIX C. BLAND ALTMAN PLOTS ESTIMATES AND ACTUAL UTILITY VALUES MODEL 1-6 VALIDATION DATASET**

**
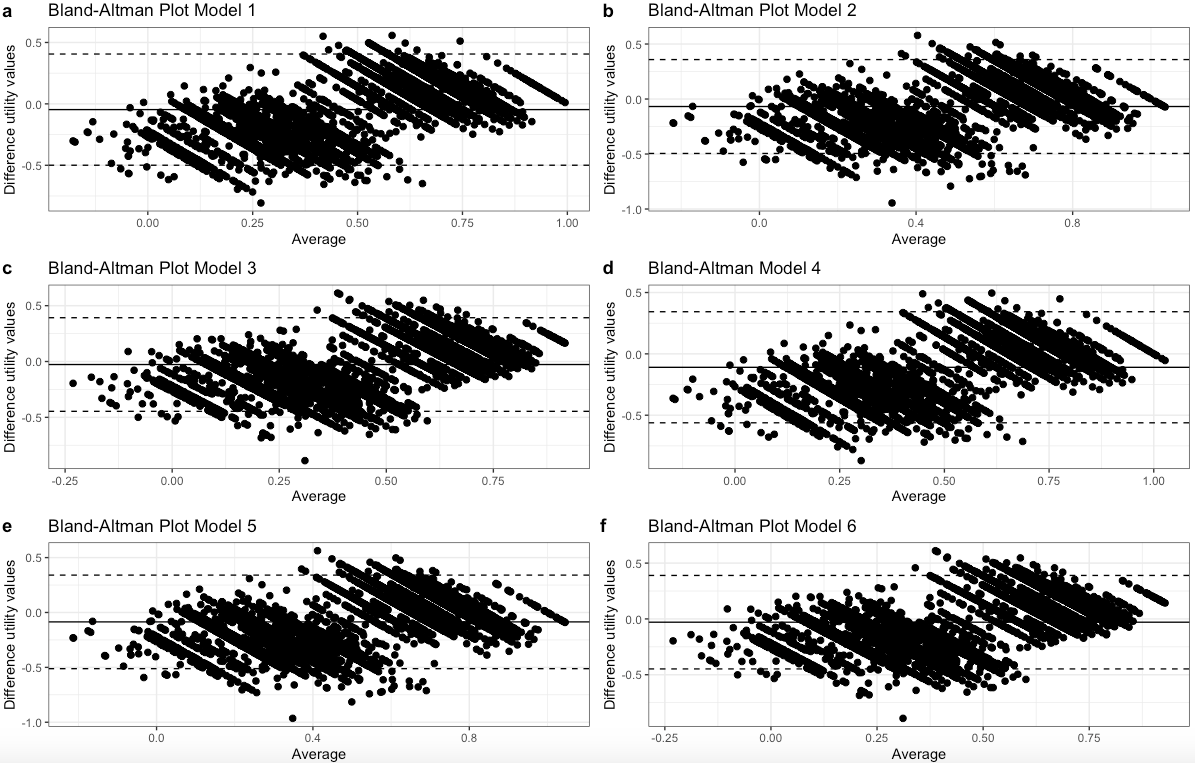
**

Legend APPENDIX C

X-axis: Average measurement of the estimated and actual utility values, Y-axis: Difference in measurements between the two instruments.

Solid line: Average difference in measurements between the estimated and actual utility values, Dashed lines: 95% confidence interval limits for the average difference.

**APPENDIX D. SENSITIVITY ANALYSIS**

| SA 1 Mental Health | with mental health | | | without mental health | | |
| --- | --- | --- | --- | --- | --- | --- |
|  | RMSE | R^2^ | AIC | RMSE | R^2^ | AIC |
| Model 1; OLS with ODI total scores | 0.21 | 0.46 | -738.80 | 0.22 | 0.43 | -617.10 |
| Model 2; OLS with ODI individual items scores (continuous) | 0.20 | 0.49 | -940.55 | 0.21 | 0.47 | -802.74 |
| Model 3; OLS with ODI individual items scores (ordered) | 0.20 | 0.52 | -1004.90 | 0.20 | 0.49 | -869.50 |
| Model 4; Tobit with ODI total scores | 0.21 | 0.48 | -699.13 | 0.22 | 0.44 | -578.63 |
| Model 5; Tobit with ODI individual items scores (continuous) | 0.20 | 0.51 | -904.42 | 0.21 | 0.48 | -765.69 |
| Model 6; Tobit with ODI individual items scores (ordered) | 0.20 | 0.52 | -960.76 | 0.20 | 0.49 | -826.14 |
| OLS: Ordinary Least Squares Regression, ODI: Oswestery Disability Index, RMSE: root-mean-square error, R2: proportion of variation in the dependent variable, AIC: Akaike information criterion | | | | | | |

| SA 2 Living with partner | with variable partner | | | without variable partner | | |
| --- | --- | --- | --- | --- | --- | --- |
|  | RMSE | R^2^ | AIC | RMSE | R^2^ | AIC |
| Model 1; OLS with ODI total scores | 0.22 | 0.45 | -2326.48 | 0.22 | 0.45 | -2318.62 |
| Model 2; OLS with ODI individual items scores (continuous) | 0.21 | 0.50 | -3423.24 | 0.21 | 0.50 | -3401.74 |
| Model 3; OLS with Stepwise Selection AIC with ODI sub scores (ordered) | 0.21 | 0.51 | -3768.53 | 0.21 | 0.51 | -3762.27 |
| Model 4; Tobit with ODI total scores | 0.22 | 0.46 | -2054.46 | 0.22 | 0.46 | -2061.91 |
| Model 5; Tobit with Stepwise Selection AIC with ODI sub scores (continuous) | 0.21 | 0.50 | -3155.95 | 0.21 | 0.50 | -3164.37 |
| Model 6; Tobit with Stepwise Selection AIC with ODI sub scores (ordered) | 0.21 | 0.51 | -3467.56 | 0.21 | 0.51 | -3473.60 |
| OLS: Ordinary Least Squares Regression, ODI: Oswestery Disability Index, RMSE: root-mean-square error, R2: proportion of variation in the dependent variable, AIC: Akaike information criterion | | | | | | |

| SA3 Cross walk EQ-5D-3 | EQ-5D-3L | | | EQ-5D-5L reversed cross walk | | |
| --- | --- | --- | --- | --- | --- | --- |
|  | RMSE | R^2^ | AIC | RMSE | R^2^ | AIC |
| Model 1; OLS with ODI total scores | 0.22 | 0.45 | -2326.48 | 0.15 | 0.49 | -12150.58 |
| Model 2; OLS with ODI individual items scores (continuous) | 0.21 | 0.50 | -3423.24 | 0.15 | 0.53 | -13158.25 |
| Model 3; OLS with Stepwise Selection AIC with ODI sub scores (ordered) | 0.21 | 0.51 | -3769.51 | 0.14 | 0.54 | -13412.33 |
| Model 4; Tobit with ODI total scores | 0.22 | 0.45 | -2061.91 | 0.15 | 0.49 | -12156.93 |
| Model 5; Tobit with Stepwise Selection AIC with ODI sub scores (continuous) | 0.21 | 0.50 | -3164.37 | 0.15 | 0.53 | -13158.25 |
| Model 6; Tobit with Stepwise Selection AIC with ODI sub scores (ordered) | 0.21 | 0.51 | -3474.88 | 0.14 | 0.54 | -13412.33 |
| OLS: Ordinary Least Squares Regression, ODI: Oswestery Disability Index, RMSE: root-mean-square error, R2: proportion of variation in the dependent variable, AIC: Akaike information criterion | | | | | | |

**APPENDIX E. BLAND ALTMAN PLOTS ESTIMATED AND ACTUAL UTILITY VALUES MODEL 1-6 EMPIRICAL DATASETS**

**
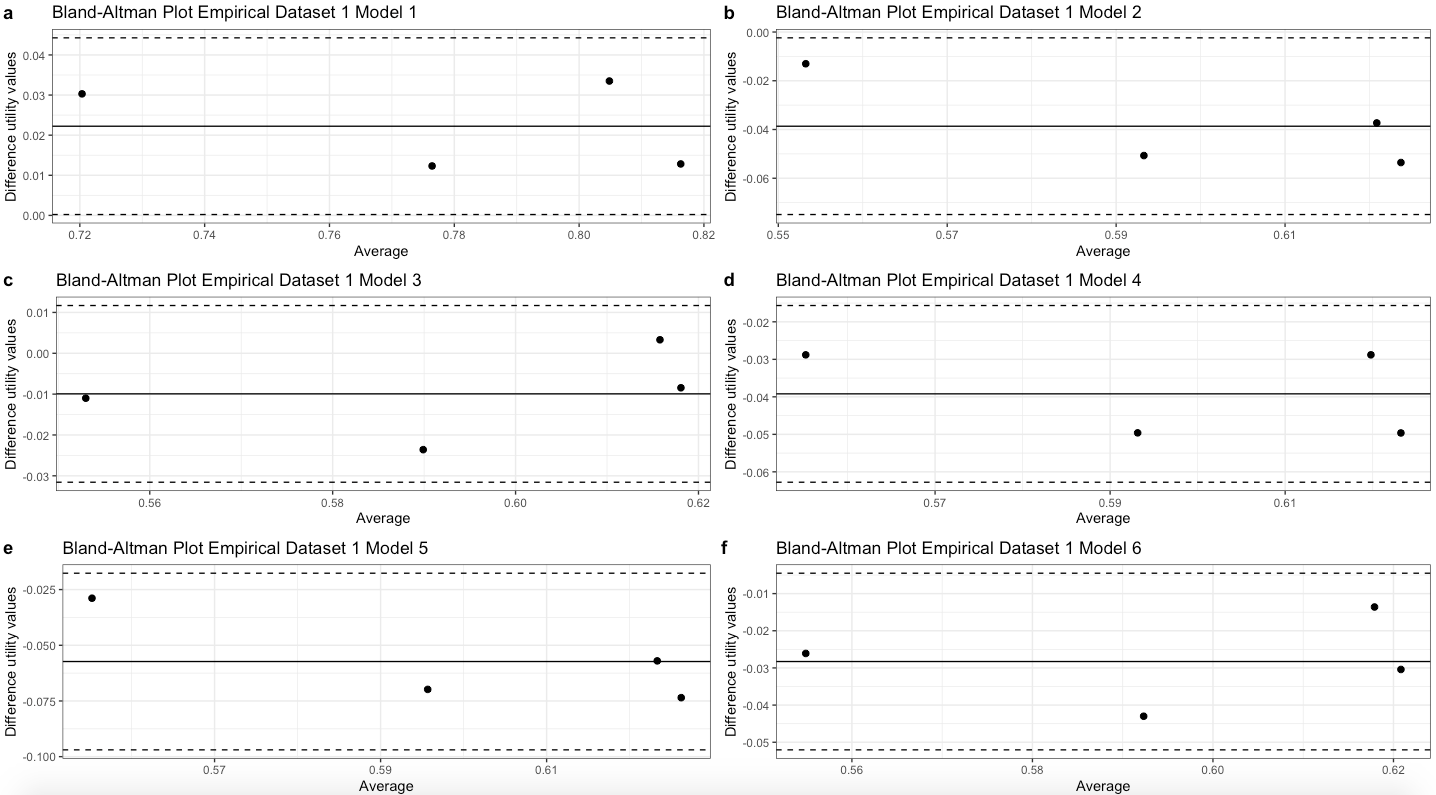
**

.

**
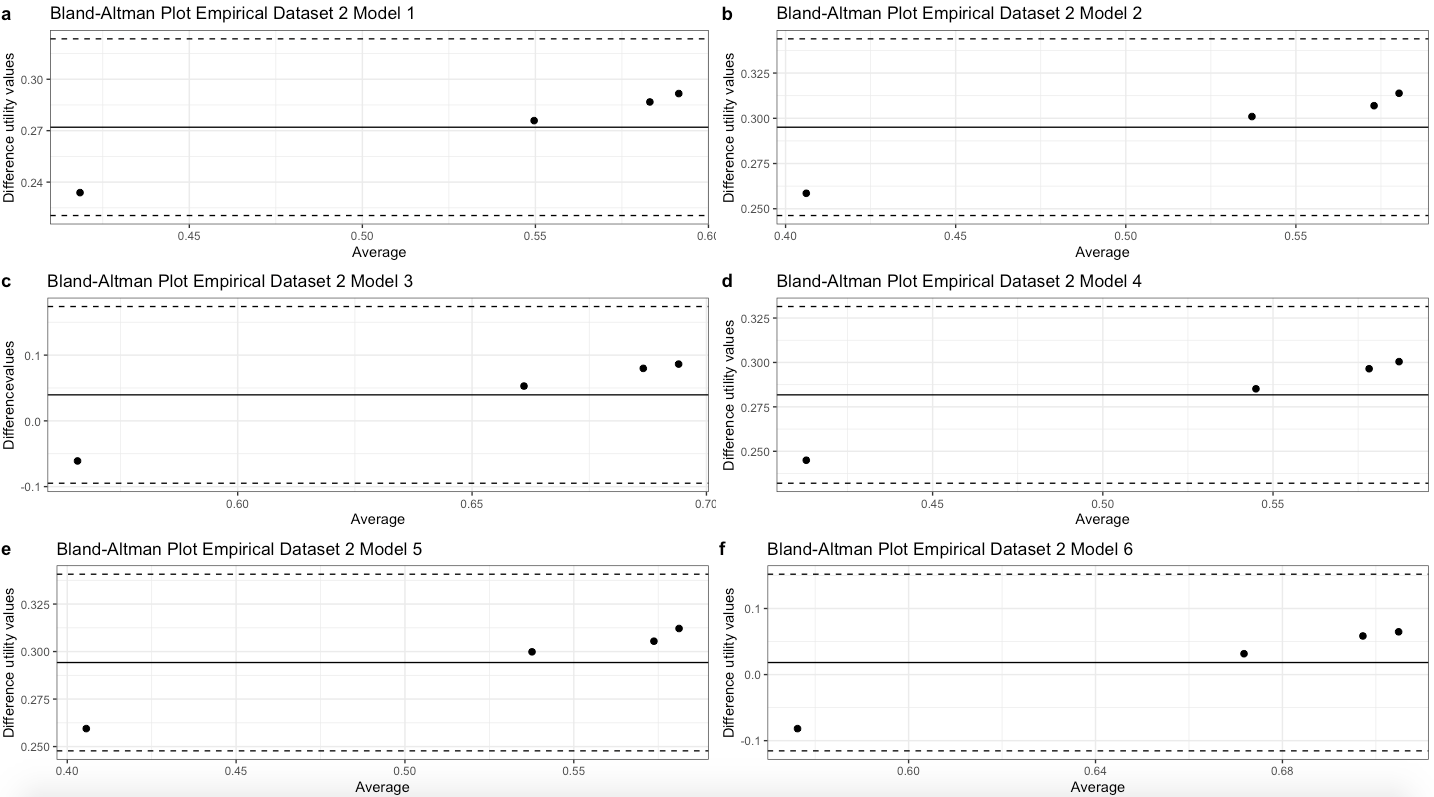
**

Legend APPENDIX E

X-axis: Average measurement of the estimated and actual utility values, Y-axis: Difference in measurements between the two instruments.

Solid line: Average difference in measurements between the estimated and actual utility values, Dashed lines: 95% confidence interval limits for the average difference.

**APPENDIX F. REGRESSION COEFFICIENTS PER COUNTRIE FOR MODEL 2 AND 5**

| **Regression coefficients Model 2** | | | | | | | | |
| --- | --- | --- | --- | --- | --- | --- | --- | --- |
|  | UK | Spain | Japan | Zimbabwe | Germany | USA | South Korea | Denmark |
| Intercept | 0.9216145 | 0.9629999 | 0.8061 | 0.8922 | 1.0250294 | 0.9124882 | 0.9344 | 0.8973506 |
| ODI1 | -0.1048651 | -0.0912104 | -0.03095 | -0.05299 | -0.0975522 | -0.0648293 | -0.03990 | -0.0757004 |
| ODI2 | -0.0527377 | -0.0623234 | -0.02753 | -0.03930 | -0.0437269 | -0.0386020 | -0.03050 | -0.0398935 |
| ODI3 | -0.0066237 | -0.0083612 | -0.003670 | -0.004353 | -0.0052832 | -0.0044403 | -0.004936 | -0.0045352 |
| ODI4 | -0.0261759 | -0.0311805 | -0.01816 | -0.01697 | -0.0254695 | -0.0176813 | -0.02332 | -0.0237435 |
| ODI5 | -0.0052813 | -0.0059330 | -0.004216 | -0.003785 | -0.0032939 | -0.0043486 | -0.004155 | -0.0056333 |
| ODI6 | -0.0114667 | -0.0124294 | -0.005125 | -0.006387 | -0.0125239 | -0.0070242 | -0.006542 | -0.0068254 |
| ODI7 | -0.0144788 | -0.0125305 | -0.005614 | -0.008621 | -0.0119149 | -0.0095046 | -0.006776 | -0.0132994 |
| ODI9 | -0.0338144 | -0.0360919 | -0.01856 | -0.02075 | -0.0246979 | -0.0230226 | -0.02228 | -0.0272122 |
| ODI10 | -0.0220272 | -0.0247321 | -0.01189 | -0.01395 | -0.0167978 | -0.0154409 | -0.01668 | -0.0190554 |
| Age | 0.0017108 | 0.0016504 | 0.0007201 | 0.0009132 | 0.0012912 | 0.0011823 | 0.0008650 | 0.0013902 |
| Sex; female | 0.0082351* | 0.0127427 | 0.009772 | 0.007933 |  | 0.0078843 | 0.01142 | 0.0095676 |
| Education; middle | 0.0162531 | 0.0158689 | 0.007196 | 0.009566 | 0.0112980 | 0.0111634 | 0.009091 | 0.0129300 |
| Education; high | 0.0218355 | 0.0219954 | 0.01072 | 0.01294 | 0.0141464 | 0.0148199 | 0.01210 | 0.0153532 |
| No partner | -0.0113913 | -0.0085004* | -0.004576 | -0.005383 | -0.0072071 * | -0.0088351 | -0.003601 | -0.0107631 |
| Secondary care | -0.0820832 | -0.0842040 | -0.05519 | -0.04963 | -0.0788964 | -0.0492312 | -0.04828 | -0.0481359 |
| Tertiary care | -0.0656978 | -0.0748289 | -0.05581 | -0.04609 | -0.0620024 | -0.0411599 | -0.04427 | -0.0364969 |
| NRS; moderate | 0.0388160 | 0.0359657 | 0.004944 * | 0.01767 | 0.0395182 | 0.0239697 | 0.01842 | 0.0322145 |
| NRS; severe | -0.0666840 | -0.0567207 | -0.01999 | -0.03398 | -0.0612173 | -0.0416691 | -0.01741 | -0.0391295 |
| R^2^ model | 0.5155 | 0.5427 | 0.5339 | 0.5631 | 0.4884 | 0.5289 | 0.5341 | 0.5173 |
| * not significant | | | | | | | | |

| **Regression coefficients Model 2** | | | | | | | | |
| --- | --- | --- | --- | --- | --- | --- | --- | --- |
|  | France | Thailand | Canada | China | Italy | Singapore | Taiwan | Argentina |
| Intercept | 0.8920794 | 0.8011386 | 0.8843 | 0.8854 | 0.9449 | 0.8237519 | 0.7539217 | 0.9289 |
| ODI1 | -0.0698340 | -0.0586726 | -0.05749 | -0.04194 | -0.05321 | -0.0802764 | -0.0730139 | -0.05980 |
| ODI2 | -0.0734759 | -0.0487556 | -0.03653 | -0.04289 | -0.03633 | -0.0687301 | -0.0657758 | -0.02262 |
| ODI3 | -0.0081591 | -0.0058457 | -0.003727 | -0.005300 | -0.004218 | -0.0102091 | -0.0081129 | -0.004647 |
| ODI4 | -0.0279455 | -0.0252811 | -0.01788 | -0.01989 | -0.02106 | -0.0302459 | -0.0267910 | -0.02291 |
| ODI5 | -0.0059446 | -0.0047299 | -0.004710 | -0.003938 | -0.005428 | -0.0068003 | -0.0055472 | -0.004378 |
| ODI6 | -0.0131530 | -0.0103183 | -0.005809 | -0.007129 | -0.004731 | -0.0153680 | -0.0110460 | -0.01036 |
| ODI7 | -0.0119208 | -0.0090852 | -0.01049 | -0.007816 | -0.009086 | -0.0121587 | -0.0120211 | -0.007826 |
| ODI9 | -0.0366947 | -0.0248885 | -0.02213 | -0.02582 | -0.02105 | -0.0482907 | -0.0404785 | -0.01948 |
| ODI10 | -0.0176721 | -0.0153155 | -0.01320 | -0.01513 | -0.01709 | -0.0212139 | -0.0224594 | -0.01225 |
| Age | 0.0012969 | 0.0009576 | 0.001065 | 0.0008914 | 0.001171 | 0.0015746 | 0.0014902 | 0.0007053 |
| Sex; female | 0.0143939 | 0.0104631 | 0.007273 | 0.01227 | 0.009246 | 0.0151897 | 0.0177048 | 0.006471 |
| Education; middle | 0.0188068 | 0.0105884 | 0.01101 | 0.01318 | 0.007618 | 0.0255564 | 0.0215434 | 0.006118 |
| Education; high | 0.0325035 | 0.0162018 | 0.01475 | 0.02090 | 0.006850* | 0.0457172 | 0.0349359 | 0.007121 * |
| No partner | -0.0100189 | -0.0056038 * | -0.009769 | -0.005817 | -0.007207 | -0.0126894 | -0.0109666 |  |
| Secondary care | -0.1155225 | -0.0892417 | -0.04946 | -0.06664 | -0.02561 * | -0.1584970 | -0.1102171 | -0.09597 |
| Tertiary care | -0.1209327 | -0.0879129 | -0.04343 | -0.06882 | -0.02004* | -0.1554560 | -0.1116358 | -0.07828 |
| NRS; moderate | 0.0080468 * | 0.0131317 | 0.01803 | 0.008445 | 0.02737 | -0.0012864* | 0.0126286* | 0.01409 |
| NRS; severe | -0.0622104 | -0.0425631 | -0.03582 | 0.03084 | -0.02272 | -0.0773060 | -0.0584446 | -0.03883 |
| R^2^ Model | 0.5757 | 0.5655 | 0.533 | 0.5753 | 0.515 | 0.557 | 0.5611 | 0.4742 |
| * not significant | | | | | | | | |

| **Regression coefficients Model 2** | | | | | | | | |
| --- | --- | --- | --- | --- | --- | --- | --- | --- |
|  | Australia | Brazil | Chile | Hungary | Poland | Portugal | Sri Lanka | Sweden |
| Intercept | 0.9025891 | 0.7976 | 0.8235944 | 0.9472674 | 0.9930241 | 0.7336 | 0.8370042 | 0.9092 |
| ODI1 | -0.0801515 | -0.02961 | -0.0632935 | -0.0601439 | -0.0920958 | -0.04206 | -0.0599403 | -0.02571 |
| ODI2 | -0.0435985 | -0.04156 | -0.0591976 | -0.0446507 | -0.0406323 | -0.05540 | -0.0582604 | -0.01638 |
| ODI3 | -0.0046462 | -0.005133 | -0.0081320 | -0.0053581 | -0.0055945 | -0.007265 | -0.0084620 | -0.003047 |
| ODI4 | -0.0209143 | -0.02042 | -0.0271107 | -0.0283114 | -0.0253303 | -0.02402 | -0.0407823 | -0.01112 |
| ODI5 | -0.0040522 | -0.004258 | -0.0058750 | -0.0073975 | -0.0052011 | -0.006597 | -0.0059588 | -0.002437 |
| ODI6 | -0.0087226 | -0.007792 | -0.0099633 | -0.0039895 | -0.0089339 | -0.009148 | -0.0134058 | -0.004826 |
| ODI7 | -0.0118253 | -0.005723 | -0.0108634 | -0.0110644 | -0.0127106 | -0.007490 | -0.0106433 | -0.004880 |
| ODI9 | -0.0287889 | -0.02157 | -0.0375882 | -0.0273724 | -0.0271976 | -0.02720 | -0.0351063 | -0.01726 |
| ODI10 | -0.0169747 | -0.01159 | -0.0238120 | -0.0257401 | -0.0217994 | -0.01842 | -0.0248097 | -0.007187 |
| Age | 0.0013476 | 0.0006081 | 0.0013849 | 0.0015927 | 0.0014433 | 0.0009556 | 0.0012154 | 0.005130 |
| Sex; female | 0.0072436 | 0.01137 | 0.0170097 | 0.0144952 | 0.0063456* | 0.01633 | 0.0191124 | 0.005607 |
| Education; middle | 0.0134860 | 0.009266 | 0.0180989 | 0.0098368 | 0.0104315 | 0.009909 | 0.0139406 | 0.009827 |
| Education; high | 0.0202586 | 0.01642 | 0.0289852 | 0.0064924* | 0.0103657 | 0.01632 | 0.0208065 | 0.01747 |
| No partner | -0.0110786 | -0.003376* | -0.0083945 | -0.0085097 | -0.0064591* | -0.08909 |  | -0.005084 |
| Secondary care | -0.0705204 | -0.08020 | 0.0893192 |  | -0.0467255 | -0.09395 | -0.0996952 | -0.05385 |
| Tertiary care | -0.0601057 | -0.08623 | -0.0882755 |  | -0.0273513* | 0.003993* | -0.0989189 | -0.05025 |
| NRS; moderate | 0.0255881 | -0.002747* | 0.0146376 | 0.0403118 | 0.0443564 | 0.005330 | 0.0213804 | -0.0009108* |
| NRS; severe | -0.0541719 | -0.02768 | -0.0452285 | -0.0159125 | -0.0466466 | -0.03400 | -0.0330848 | -0.02.275 |
| R^2^ model | 0.53 | 0.5747 | 0.5681 | 0.4754 | 0.5109 | 0.5689 | 0.552 | 0.5174 |
| * not significant | | | | | | | | |

| **Regression coefficients Model 2** | | | | | | | |
| --- | --- | --- | --- | --- | --- | --- | --- |
|  | Trinidad Tobago | Belgium | Finland | Iran | Malaysia | New Zealand | Slovenia |
| Intercept | 0.8883515 | 0.8447444 | 0.7394 | 0.7589 | 0.9021951 | 0.8058 | 0.7929 |
| ODI1 | -0.0390160 | -0.0675222 | -0.01883 | -0.03214 | -0.0403614 | -0.05954 | -0.03393 |
| ODI2 | -0.0307738 | -0.0396007 | -0.03637 | -0.06078 | -0.0378891 | -0.03396 | -0.03881 |
| ODI3 | -0.0035665 | -0.0051525 | -0.003874 | -0.006695 | -0.0047368 | -0.004221 | -0.004225 |
| ODI4 | -0.0176425 | -0.0182410 | -0.01274 | -0.01571 | -0.0162989 | -0.01650 | -0.02606 |
| ODI5 | -0.0046815 | -0.0031076 | -0.003825 | -0.005023 | -0.0029200 | -0.002557* | -0.002369 |
| ODI6 | -0.0048159 | -0.0086009 | -0.003055 | -0.007477 | -0.0069253 | -0.007854 | -0.01007 |
| ODI7 | -0.0071148 | -0.0096681 | -0.005288 | -0.007503 | -0.0060276 | -0.008642 | -0.007424 |
| ODI9 | -0.0154423 | -0.0282179 | -0.02356 | -0.03182 | -0.0224816 | -0.02426 | -0.02483 |
| ODI10 | -0.0122454 | -0.0153905 | -0.01171 | -0.01354 | -0.0123636 | -0.01277 | -0.01078 |
| Age | 0.0008015 | 0.0011642 | 0.0007940 | 0.0009362 | 0.0007950 | 0.0009665 | 0.0004864 |
| Sex; female | 0.0076287 | 0.0087419 | 0.01435 | 0.01686 | 0.0095461 | 0.007549 | 0.01211 |
| Education; middle | 0.0048550 | 0.0159546 | 0.01307 | 0.01732 | 0.0114116 | 0.01397 | 0.01465 |
| Education; high | 0.0045773* | 0.0248290 | 0.02222 | 0.03269 | 0.0190117 | 0.02191 | 0.02579 |
| No partner | -0.0042406 | -0.0097974 | -0.008083 | -0.007903 | -0.0053342 | -0.008842 | -0.006400 |
| Secondary care | -0.0347116 | -0.0818253 | -0.05806 | -0.09298 | -0.0636079 | -0.08000 | -0.1092 |
| Tertiary care | -0.0323348 | -0.0744276 | -0.06886 | -0.01078 | -0.0650069 | -0.07378 | -0.1153 |
| NRS; moderate | 0.0152583 | 0.0165661 | -0.007442 | -0.01283 | 0.0077327 * | 0.01226 | -0.003784* |
| NRS; severe | -0.0196063 | -0.0503878 | -0.02146 | -0.04328 | -0.0327250 | -0.04561 | -0.03126 |
| R2 | 0.5291 | 0.5195 | 0.471 | 0.5374 | 0.5658 | 0.5176 | 0.5395 |
| * not significant | | | | | | | |

| **Regression coefficients Model 5** | | | | | | | | |
| --- | --- | --- | --- | --- | --- | --- | --- | --- |
|  | UK | Spain | Japan | Zimbabwe | Germany | USA | South Korea | Denmark |
| Intercept 1 | 0.9472972 | 0.9895059 | 0.8120 | 0.9032 | 1.0534441 | 0.9276111 | 0.9454 | 0.9154778 |
| Intercept 2 | -1.4601898 | -1.5052735 | -2.332 | -2.084 | -1.5363619 | -1.9010432 | -2.118 | -1.7364138 |
| ODI1 | -0.1060945 | -0.0924513 | -0.03121 | -0.05350 | -0.0988903 | -0.0655513 | -0.04043 | -0.0765778 |
| ODI2 | -0.0524924 | -0.0620764 | -0.02748 | -0.03920 | -0.0434683 | -0.0384583 | -0.03037 | -0.0397189 |
| ODI3 | -0.0068076 | -0.0085525 | -0.003711 | -0.004431 | -0.0054762 | -0.0045482 | -0.005017 | -0.0046644 |
| ODI4 | -0.0261440 | -0.0311491 | -0.01815 | -0.01696 | -0.0254331 | -0.0176635 | -0.02340 | -0.0237235 |
| ODI5 | -0.0056060 | -0.0062683 | -0.004289 | -0.003924 | -0.0036473* | -0.0045421 | -0.004265 | -0.0058678 |
| ODI6 | -0.0118429 | -0.0128131 | -0.005210 | -0.006547 | -0.0129204 | -0.0072481 | -0.006690 | -0.0070971 |
| ODI7 | -0.0144099 | -0.0124585 | -0.00560 | -0.008593 | -0.0118338 | -0.0094637 | -0.006740 | -0.0132512 |
| ODI9 | -0.0342032 | -0.0364879 | -0.01864 | -0.02092 | -0.0251148 | -0.0232536 | -0.02251 | -0.0274933 |
| ODI10 | -0.0218918 | -0.0245948 | -0.01186 | -0.01389 | -0.0166507 | -0.0153614 | -0.01667 | -0.0189594 |
| Age | 0.0016847 | 0.0016240 | 0.0007145 | 0.0009022 | 0.0012619 | 0.0011671 | 0.0008651 | 0.0013719 |
| Sex; female | 0.0085295 | 0.0130419 | 0.009837 | 0.008057 |  | 0.0080605 | 0.01135 | 0.0097820 |
| Education; middle | 0.0159807 | 0.0155870 | 0.007139 | 0.009451 | 0.0110166 | 0.0110042 | 0.008913 | 0.0127378 |
| Education; high | 0.0219722 | 0.0221218 | 0.01075 | 0.01299 | 0.0142850 | 0.0149028 | 0.01213 | 0.0154533 |
| No partner | -0.0111703 | -0.0082751* | -0.004524 | -0.005286 | -0.0069407* | -0.0087042 |  | -0.0106022 |
| Secondary care | -0.0960691 | -0.0988243 | -0.05854 | -0.05567 | -0.0944005 | -0.0574566 | -0.05516 | -0.0579199 |
| Tertiary care | -0.0792959 | -0.0890656 | -0.05907 | -0.05197 | -0.0771188 | -0.0491595 | -0.05105 | -0.0460041 |
| NRS; moderate | 0.0336000 | 0.0306813 | 0.003827* | 0.01548 | 0.0338432 | 0.0208932 | 0.01602 | -0.0460041 |
| NRS; severe | -0.0708414 | -0.0609302 | -0.02088 | -0.03573 | -0.0657410 | -0.0441208 | -0.01939 | -0.0420809 |
| R^2^ model | 0.5154651 | 0.5427006 | 0.533909 | 0.5630955 | 0.4882995 | 0.5288496 | 0.533964 | 0.5172503 |
| * not significant | | | | | | | | |

| **Regression coefficients Model 5** | | | | | | | | |
| --- | --- | --- | --- | --- | --- | --- | --- | --- |
|  | France | Thailand | Canada | China | Italy | Singapore | Taiwan | Argentina |
| Intercept 1 | 0.9105936 | 0.8118602 | 0.8965 | 0.8959 | 0.9608 | 0.8432469 | 0.7693004 | 0.9428 |
| Intercept 2 | -1.6470017 | -1.9013025 | -1.997 | -2.098 | -1.973 | -1.5005430 | -1.6125055 | -1.924 |
| ODI1 | -0.0706407 | -0.0591457 | -0.05807 | -0.04240 | -0.05398 | -0.0811177 | -0.0736964 | -0.06042 |
| ODI2 | -0.0733158 | -0.0486618 | -0.03641 | -0.04280 | -0.03618 | -0.0685617 | -0.0656403 | -0.02250 |
| ODI3 | -0.0082881 | -0.0059205 | -0.003641 | -0.005374 | -0.004334 | -0.0103438 | -0.0082196 | -0.004745 |
| ODI4 | -0.0279223 | -0.0252677 | -0.01786 | -0.01988 | -0.02105 | -0.0302196 | -0.0267717 | -0.02288 |
| ODI5 | -0.0061698 | -0.0048615 | -0.004866 | -0.004068 | -0.005639 | -0.0070336 | -0.0057366 | -0.004546 |
| ODI6 | -0.0134090 | -0.0104702 | -0.005989 | -0.007278 | -0.004974 | -0.0156361 | -0.0112651 | -0.01055 |
| ODI7 | -0.0118732 | -0.0090583 | -0.01045 | -0.007789 | -0.009042 | -0.0121089 | -0.0119813 | -0.007793 |
| ODI9 | -0.0369600 | -0.0250451 | -0.02231 | -0.02597 | -0.02130 | -0.0485695 | -0.0407062 | -0.01968 |
| ODI10 | -0.0175793 | -0.0152620 | -0.01314 | -0.01508 | -0.01700 | -0.0211177 | -0.0223827 | -0.01218 |
| Age | 0.0012792 | 0.0009472 | 0.001053 | 0.0008813 | 0.001155 | 0.0015561 | 0.0014754 | 0.0006913 |
| Sex; female | 0.0145922 | 0.0105793 | 0.007414 | 0.01238 | 0.009436 | 0.0153978 | 0.0178762 | 0.006627 |
| Education; middle | 0.0186230 | 0.0104814 | 0.01088 | 0.01307 | 0.007445 | 0.0253681 | 0.0213923 | 0.005979* |
| Education; high | 0.0325879 | 0.0162499 | 0.01482 | 0.02095 | 0.006931* | 0.0458146 | 0.0350178 | 0.007173* |
| No partner | -0.0098760 | -0.0055124* | -0.009664 | -0.005731 | -0.007059 | -0.0125426 | -0.0108399 |  |
| Secondary care | -0.1261291 | -0.0953430 | -0.05617 | -0.07265 | -0.03417 | -0.1697453 | -0.1189331 | -0.1040 |
| Tertiary care | -0.1312972 | -0.0938662 | -0.04996 | -0.07468 | -0.02836 | -0.1664425 | -0.1201396 | -0.08606 |
| NRS; moderate | 0.0045179* | 0.0110833 | 0.01556 | 0.006422* | 0.02409 | -0.0049764* | 0.0096595* | 0.01145 |
| NRS; severe | -0.0650354 | -0.0441987 | -0.03779 | -0.03246 | -0.02532 | -0.0802601 | -0.0608164 | -0.04093 |
| R^2^ model | 0.5756387 | 0.565525 | 0.5329835 | 0.57526 | 0.51494 | 0.5570274 | 0.5610422 | 0.4741354 |
| * not significant | | | | | | | | |

| **Regression coefficients Model 5** | | | | | | | | |
| --- | --- | --- | --- | --- | --- | --- | --- | --- |
|  | Australia | Brazil | Chile | Hungary | Poland | Portugal | Sri Lanka | Sweden |
| Intercept 1 | 0.9205291 | 0.8039 | 0.8390576 | 0.9594831 | 1.0188753 | 0.7424590 | 0.854156 | 0.9092 |
| Intercept 2 | -1.7247032 | -2.244 | -1.7118986 | -1.6827221 | -1.6065257 | -1.9445409 | -1.648065 | -2.580 |
| ODI1 | -0.0810006 | -0.02987 | -0.0639867 | -0.0613315 | -0.0933810 | -0.0424434 | -0.060690 | -0.02571 |
| ODI2 | -0.0434293 | -0.04151 | -0.0590597 | -0.0443819 | -0.0403758 | -0.0553214 | -0.058113 | -0.01638 |
| ODI3 | -0.0047727 | -0.005176 | -0.0082410 | -0.0055609 | -0.0057844 | -0.0073265 | -0.008583 | -0.003047 |
| ODI4 | -0.0208922 | -0.02042 | -0.0270919 | -0.0283079 | -0.0252997 | -0.0240094 | -0.040758 | -0.01112 |
| ODI5 | -0.0042776 | -0.004334 | -0.0060682 | -0.0077569 | -0.0055373 | -0.0067070 | -0.006172 | -0.002347 |
| ODI6 | -0.0089840 | -0.007880 | -0.0101857 | -0.0043967 | -0.0093227 | -0.0092754 | -0.013650 | -0.004826 |
| ODI7 | -0.0117777 | -0.005708 | -0.0108226 | -0.0110356 | -0.0126399 | -0.0074682 | -0.010600 | -0.004880 |
| ODI9 | -0.0290600 | -0.02166 | -0.0378188 | -0.0277944 | -0.0275985 | -0.0273258 | -0.035355 | -0.01726 |
| ODI10 | -0.0168807 | -0.01156 | -0.0237348 | -0.0256004 | -0.0216604 | -0.0183716 | -0.024724 | -0.007187 |
| Age | 0.0013295 | 0.0006022 | 0.0013700 | 0.0015644 | 0.0014163 | 0.0009470 | 0.001199 | 0.0005130 |
| Sex; female | 0.0074489 | 0.01144 | 0.0171836 | 0.0147854 | 0.0066487* | 0.0164295 | 0.019308 | 0.005607 |
| Education; middle | 0.0132987 | 0.009206 | 0.0179436 | 0.0095554 | 0.0101440 | 0.0098232 | 0.013770 | 0.009827 |
| Education; high | 0.0203570 | 0.01645 | 0.0290658 | 0.0066940* | 0.0104967 | 0.0163578 |  | 0.01742 |
| No partner | -0.0109283 | -0.003323* | -0.0082643 | -0.0082528 | -0.0062182* |  | 0.020878 | -0.005084 |
| Secondary care | -0.0803519 | -0.08387 | -0.0980271 |  | -0.0604633 | -0.0941681 | -0.109474 | -0.05385 |
| Tertiary care | -0.0696706 | -0.08982 | -0.0967684 |  | -0.0406806* | -0.0989100 | -0.108462 | -0.05025 |
| NRS; moderate | 0.0219669 | -0.003909* | 0.0116307* | 0.0353516 | 0.0389747 | 0.0023267* | 0.018152 | -0.0009108* |
| NRS; severe | -0.0570602 | -0.02861 | -0.0476263 | -0.0198679 | -0.0509219 | -0.0353305 | -0.035651 | -0.02275 |
| R^2^ model | 0.5300021 | 0.5746986 | 0.5680596 | 0.475328 | 0.510803 | 0.5688545 | 0.5519598 | 0.5174331 |
| * not significant | | | | | | | | |

| **Regression coefficients Model 5** | | | | | | | |
| --- | --- | --- | --- | --- | --- | --- | --- |
|  | Trinidad Tobago | Belgium | Finland | Iran | Malaysia | New Zealand | Slovenia |
| Intercept 1 | 0.8972 | 0.8585886 | 0.7453 | 0.7684 | 0.9119 | 0.8161 | 0.8008 |
| Intercept 2 | -2.253 | -1.8174829 | -2.232 | -1.943 | -2.191 | -1.952 | -2.091 |
| ODI1 | -0.03942 | -0.0681537 | -0.01908 | -0.03253 | -0.04079 | -0.06001 | -0.03424 |
| ODI2 | -0.03069 | -0.0394748 | -0.03632 | -0.06070 | -0.03780 | -0.03387 | -0.03875 |
| ODI3 | -0.003629 | -0.0052490 | -0.003914 | -0.006760 | -0.004805 | -0.004292 | -0.004277 |
| ODI4 | -0.01763 | -0.0182228 | -0.01273 | -0.01570 | -0.01629 | -0.01649 | -0.02604 |
| ODI5 | -0.004795 | -0.0032777 | -0.003899 | -0.005138 | -0.003039 | -0.002683* | -0.002459 |
| ODI6 | -0.004947 | -0.0087993 | -0.003142 | -0.007610 | -0.007062 | -0.008001 | -0.01018 |
| ODI7 | -0.007092 | -0.009632 | -0.005273 | -0.007479 | -0.006002 | -0.008615 | -0.007406 |
| ODI9 | -0.01558 | -0.0284241 | -0.02365 | -0.03196 | -0.02262 | -0.02441 | -0.02494 |
| ODI10 | -0.01220 | -0.0153200 | -0.01168 | -0.01349 | -0.01232 | -0.01272 | -0.01074 |
| Age | 0.0007929 | 0.0011506 | 0.0007885 | 0.0009273 | 0.0007857 | 0.0009564 | 0.0004793 |
| Sex; female | 0.007729 | 0.0088975 | 0.01442 | 0.01697 | 0.009653 | 0.007664 | 0.01219 |
| Education; middle | 0.004763 | 0.0158167 | 0.01301 | 0.01723 | 0.01132 | 0.01387 | 0.01458 |
| Education; high | 0.004618* | 0.0249067 | 0.02225 | 0.03275 | 0.01906 | 0.02197 | 0.02583 |
| No partner | -0.004159* | -0.0096840 | -0.008030 | -0.007824 | -0.005256 | -0.008757 | -0.006341 |
| Secondary care | -0.03957 | -0.0895791 | -0.06151 | -0.09850 | -0.06916 | -0.08582 | -0.1138 |
| Tertiary care | -0.03706 | -0.0819805 | -0.07223 | -0.1132 | -0.07043 | -0.07945 | -0.1198 |
| NRS; moderate | 0.01351 | 0.0138492 | -0.008571 | -0.01461 | 0.005869* | 0.01026* | -0.005173* |
| NRS; severe | -0.02100 | -0.0525574 | -0.02236 | -0.04471 | -0.03241 | -0.04722 | -0.03237 |
| R^2^ model | 0.5290929 | 0.5194592 | 0.4709515 | 0.5374136 | 0.5657962 | 0.5176076 | 0.5394758 |
| * not significant | | | | | | | |
